# GWAS for behavioral traits in golden retrievers identifies genes implicated in human temperament, mental health, and cognition

**DOI:** 10.1073/pnas.2421757122

**Published:** 2025-11-24

**Authors:** Enoch Alex, Paul Gennotte, Anna Morros Nuevo, Yunzhu Yu, Benjamin Keep, Megan Sullivan, Daniel Mills, Varun Warrier, Eleanor Raffan

**Affiliations:** ^a^Department of Physiology, Development and Neuroscience, University of Cambridge, Cambridge CB2 3EG, United Kingdom; ^b^Department of Psychology, School of Biological and Behavioural Sciences, Queen Mary University of London, London E1 4NS, United Kingdom; ^c^Department of Biological and Life Sciences, School of Natural Sciences, University of Lincoln, Lincoln LN6 7TS, United Kingdom; ^d^Department of Psychiatry, University of Cambridge, Cambridge CB2 0SZ, United Kingdom; ^e^Department of Psychology, University of Cambridge, Cambridge CB2 3EB, United Kingdom

**Keywords:** genetics, behaviour, psychiatric, C-BARQ, dog

## Abstract

Dogs display temperamental and behavioral variation between individuals, just as psychiatric, temperamental, and cognitive traits vary in humans. In both species, these traits are highly heritable, yet causal genes remain incompletely understood. We performed 14 genome-wide association studies (GWAS) for behavioral traits quantified using the Canine Behavioral Assessment and Research Questionnaire (C-BARQ) in ~1,000 golden retrievers, identifying 12 genome-wide significant loci (*P* < 2.97 × 10^−6^) for 8 traits and 9 additional loci exceeding a suggestive threshold (*P* < 1 × 10^−5^). A human phenome-wide association study (PheWAS) showed that most of the 18 canine positional candidate genes identified were associated with one or more of 190 psychiatric, temperamental, or cognitive traits in humans (7/12 genes at genome-wide loci and 5/9 at suggestive loci). For example, a genome-wide significant locus near *PTPN1* (dog-directed aggression) overlapped with human measures of Intelligence, Educational attainment, and major depressive disorder. The gene *ROMO1* was within a genome-wide significant locus for trainability in dogs and associated with intelligence, depression, irritability, and sensitivity/hurt feelings in humans. Other genes located at genome-wide significant loci associated with behavioral, psychiatric, temperamental, or cognitive traits in both species included *PRDX1(dog-directed fear), VWA8* (touch sensitivity)*, ITPR2,* and *ADGRL2/LPHN2* (trainability), and *ADD2* (stranger-directed fear). From suggestive loci we also found cross-species associations for *HUNK, and ZC3H12C,* (dog-directed fear), *SLC35F6* and *IGSF11* (separation-related problems). These results suggest that shared genetic and molecular mechanisms underlie complex behavioral and temperamental states across species and may inform our understanding of emotional states driving undesirable behaviors in dogs.

## Significance

Dogs display temperamental and behavioral variation and possess tractable genetics, making them a compelling model for studying the genetics of psychiatric, temperamental, and cognitive traits. Using a behavioral questionnaire, we phenotyped over 1,000 golden retrievers and identified 12 genome-wide significant loci and 9 suggestive loci. Of the 18 proximal candidate genes at these loci, 12 were also associated with psychiatric, temperamental, or cognitive traits in humans. Seven of these genes were at genome-wide loci and five at suggestive loci. For example, the gene nearest the dog-directed aggression locus, *PTPN1*, is associated in humans with intelligence, cognitive performance, educational attainment, and major depressive disorder. These findings suggest shared genetic mechanisms underlying behavior across species and inform emotional states underlying canine behaviors.

Dogs are highly social animals which display complex and variable behaviors and emotional states comparable to those of humans, even if they lack some of their higher cognitive functions (1). Variation in emotional reactivity, sociability, and other behavioral traits arise from complex interactions between genetic and environmental factors, with evidence of cross-species conservation (2, 3). In humans, twin studies and large-scale genome-wide association studies (GWAS) reveal moderate-to-high heritability for psychiatric, temperamental, and cognitive phenotypes (4, 5), underscoring the biological underpinnings of these adaptive distinctions. However, the genetic basis of this variation is not well understood in humans or dogs (6–8).

Research into canine behavior has revealed substantial genetic influences, evidenced by both intrabreed variability (9, 10) and distinct interbreed behavioral profiles (11–13). Ancestry-inclusive genetic studies have made progress revealing the polygenic basis of behavioral variation in dogs and its interplay with environmental factors (14, 15). However, there remains merit in mapping behavioral traits within single breeds in which deleterious variants can exist with high frequency (16, 17) and population bottlenecks at the point of breed formation resulted in high within-breed genetic homogeneity and a long range linkage disequilibrium (LD) structure that makes mapping the genes associated with complex traits remarkably tractable (18–22). Furthermore, by restricting analyses to a single breed, we avoid issues due to covariance between morphological and behavioral traits which can lead to spurious, or suspected spurious, genetic association findings. For instance, Morrill et al. dismissed a GWAS association between being “focused in distracting situations” and *FGF5* as not biologically informative because it was driven by spurious difference in focus scores between dogs with contrasting fur type (15).

A combination of phenotypic diversity and genetic tractability means dogs have been proposed as a translational model for psychiatric genetics (12, 23, 24). Some dogs develop analogs of human psychiatric conditions including age-related canine cognitive dysfunction (CCD) which is analogous to human Alzheimer’s disease (25–27), and compulsive behaviors that parallel elements of human obsessive-compulsive spectrum disorders (28–33). Genetically manipulated canine models have also been used to study autism spectrum disorder (34, 35).

The moderate to high heritability found of a wide range of human psychiatric, temperamental, and cognitive traits (4, 5) has prompted many human genetic studies, but these are frequently stymied by the complexity of gene–environment interactions inherent in human psychology and the sample sizes required (2, 3). Nonetheless, hundreds of genetic variants have been associated with human psychiatric, temperamental, and cognitive traits, the majority with small effect size. This presents further challenges as to which to prioritize for follow-on study. In that context, orthogonal data which provide evidence that genes influence similar phenotypes in another species is valuable.

No animal will be an ideal model of a human psychiatric trait because of species differences which have been comprehensively discussed elsewhere (12, 36). For instance, many diagnoses rely on the presence of symptoms which cannot be ascertained in animals (e.g., hallucinations, guilt) or which have only approximate correlates (e.g., abnormal social behavior, fear). However, dogs still offer value as a model organism because comparative genetics may be able to corroborate the importance of genes which are found to influence related traits across both species, or to identify new neurogenetic systems relevant to people (12, 36).

The high genetic correlation between many psychiatric, temperamental, or cognitive traits in people suggests that apparently divergent phenotypic manifestations may share underlying mechanistic roots, with gene-environment interactions highly influential in determining outcome (5, 37–39). A shared genetic basis has also been observed for related dog behaviors (40). A similar phenomenon is likely to occur across species; genetic variants may confer vulnerability to developing psychiatric traits in people or distinct behaviors in dogs, but the specific outcomes will be very different given the divergent cognition, life experience, and behavioral predispositions of the two species. This remains true even for traits which have apparently clear analogues in human psychiatry such as fear/anxiety, or aggression (14, 15). Therefore, in this study, we hypothesized that genes associated with behavioral traits in dogs would be associated with human traits in the same broad psychiatric, temperamental, or cognitive domains, reflecting conserved biology between the species, but did not restrict our comparisons only to narrow canine-human phenotype pairings.

We leveraged the Golden Retriever Lifetime Study (GRLS) as a valuable single-breed cohort (41, 42) in which data on behavioral traits have been collected since 2012 using the Canine Behavioral Assessment and Research Questionnaire (C-BARQ) (43). The C-BARQ gathers owner-reported observations related to 73 “items” which can be statistically grouped to form 14 “factors” (e.g., non-social fear is made up of items relating to fear of loud noises, traffic, and windblown objects) (43). These factors are described using various terms in the literature, including personality, temperament, or behaviors (44–47). For clarity, we will refer to all of them here as behavioral traits, which include dog-directed fear, stranger-directed fear, non-social fear, trainability, and various forms of aggression (43, 48). C-BARQ has demonstrated reliability against direct behavioral measures and has been used extensively to predict the suitability of dogs for working roles (49–56) or rehoming (57, 58), to assess how environment affects temperament (10, 53, 59–63), and to understand relationships between temperament and breed, health, or behavioral disorders (64–69). It has also been used to examine the genetic basis of canine behavior using GWAS (10, 14, 15, 23, 70, 71) which have identified hundreds of genetic markers linked to genes involved in brain function and development (14, 15) and highlighted the polygenic nature of canine behavior traits. The heritability of C-BARQ traits have been reported as between 0.2 to 0.7 (9, 14, 72).

However, as with any owner-reported questionnaire, the C-BARQ is not without limitations. One key concern is the potential for subjective bias, as owners may interpret their dogs’ behaviors differently based on their personal experiences, expectations, or emotional attachment to their pets (73). Additionally, environmental variability such as differences in household structure, training practices, or social exposure can influence both the behaviors exhibited by dogs and how their owners perceive them. Despite these limitations, the C-BARQ offers several advantages that make it particularly suitable for our study. First, its validated factor structure ensures consistency across studies. Second, its scalability allows for data collection from large cohorts which is essential for GWAS but is usually unfeasible using direct behavioral testing in controlled conditions, which anyway may not capture everyday natural behaviors (43, 73).

In the present study, we mapped the genetic basis of C-BARQ behavioral traits in a cohort of golden retrievers. We hypothesized that the genetic basis of canine behavioral traits might be shared in part with human psychiatric, temperamental, or cognitive traits that have similar features. Genes implicated in GWAS for C-BARQ traits in golden retrievers were tested to determine whether they were relevant in humans. We found that many canine behavior genes were also implicated in human studies of cognitive and psychiatric traits, with commonality serving to prioritize genes of interest as those impactful in both species and shed light on the potential emotional states underlying undesirable canine behaviors.

## Results

### Study Population and C-BARQ Results.

We studied 1343 golden retriever dogs from the GRLS, a longitudinal canine cohort study from which complete C-BARQ responses and related demographic data were available. We included only mature adult dogs (range 3 to 7 y, mean age 4.8 y). Sex distribution was approximately equal and the majority were gonadectomized (676 males, 76% gonadectomized; 667 females, 90% gonadectomized). C-BARQ data were analyzed using standard methods to generate 14 behavioral traits (74). Distributions were negatively skewed for 9 traits which indicates that a large majority of dogs did not display the behavior (stranger-directed aggression, dwner-directed aggression, dog-directed aggression, dog rivalry, stranger-directed fear, dog-directed fear, non-social fear, separation-related problems, and touch sensitivity, *SI Appendix,* Fig. S1). These traits were subsequently analyzed in the GWAS on a case:control basis, in part because this can improve power to detect true genetic associations responsible for extreme phenotypes and in part because the raw values would have violated GWAS LMM assumptions and normalization was not feasible for reasons detailed in *SI Appendix*, *Supplementary Text Materials and Methods*. The remainder of the factors followed a normal distribution and were analyzed as continuous traits, except for energy level score for which we hypothesized major gene effects would be more powerfully identified by comparing the most energetic dogs with controls drawn from the rest of the population. For this trait we compared dogs in the top 10% of the distribution with controls drawn randomly from the remaining 90%. C-BARQ results are reported in *SI Appendix,* Table S2 which also lists whether sex, age, neuter status, or other variables were significant in a minimal regression model; significant variables were subsequently included as covariates in the GWAS.

### Significant GWAS Associations Were Identified for Eight C-BARQ Factor Scores.

DNA was genotyped on the Axiom™ Canine Genotyping Array Sets A and B. There were 468,649 markers in 1,187 dogs which passed quality control. GWAS was conducted using a univariate linear mixed model in Genome-wide Efficient Mixed Model Analysis (GEMMA) Software v0.98.1 (75). To determine appropriate covariates, we identified the minimal regression model for each trait and study population and included only variables which were significant predictors of outcome as covariates in the GWAS (*SI Appendix*, Table S2). Continuous trait GWAS included all 1,187 dogs. Since imbalanced case and control groups can reduce GWAS power and lead to type 1 errors, we randomly selected control dogs in order not to surpass a 1:4 (*SI Appendix*, Table S2 and *Supplementary Text Materials and Method*). Heritability analysis was implemented in Genome-wide Complex Trait Analysis (GCTA) software (76), using the GREML-LDMS approach to adjust for the influence of LD and minor allele frequency (MAF) on the estimated SNP heritability which ranged from ~5 to 69% across different traits (Table 1 and *SI Appendix*, Table S3).

**Table 1.** GWAS identified significant associations for eight C-BARQ behavioral traits

Trait	Lead SNP Chr	Lead SNP position	Lead SNP variant type	*P*-value	Effect type	Effect size	Lower boundary	Upper boundary	Proximal candidate gene	Positional candidate genes	Genomic heritability, h^2^ ± SE
**DDA**	**24**	**36839070_G/A**	**Intergenic**	**2.41E−06**	**OR**	**2.09**	**36737986**	**36933844**	***PTPN1***		50.51 ± 14.88
	5	22838173_G/A	Intergenic	1.10E−05	OR	1.95	22835142	22838173	*ZC3H12C*		7.36 ± 12.38
**Dog-directed fear**	**15**	**14837906_A/G**	**Intergenic**	**5.61E** **−** **07**	**OR**	**2.23**	**13933241**	**15420702**	***PRDX1***	*LRRC41, RAD54L, LURAP1, POMGNT1, TSPAN1. MAST2, IPP, GPBP1L1, CCDC17, NASP, MMACHC, TOE1, MUTYH, HPDL, UROD, HECTD3, EIF2B3, ZSWIM5, TMEM69,* and *P3R3URF*	
	31	26842062_C/T	Intergenic	1.35E−05	OR	0.50			*HUNK*		
**Non-social fear**	**10**	**20464746_A/G**	**Intergenic**	**4.26E** **−** **06**	**OR**	**0.40**	**20382051**	**20506103**	***FBLN1***	*ATXN10* and *FBLN1*	65.61 ± 13.15
	12	58861652_T/C	Intronic	1.54E−05	OR	0.20			*ASCC3*	*ASCC3*	
	**20**	**24642572_A/G**	**Intergenic**	**6.61E** **−** **06**	**OR**	**3.20**			***KBTBD8***		
**Stranger directed fear**	7	67887313_C/A	Intergenic	2.23E−05	OR	0.06	66388377	69121669	*ADCYAP1*	*SNRPD1, ESCO1, GREB1L, ROCK1, USP14, THOC1, COLEC12, CETN1, CLUL1, TYMS, ENOSF1,* and *YES1*	19.38 ± 10.1
	**10**	**69081495_C/A**	**Intronic**	**1.44E** **−** **06**	**OR**	**2.83**	**68695939**	**69188338**	***ADD2***	*FIGLA, CLEC4F, TGFA, SNRPG, PCYOX1, TIA1,* and *C10H2orf42*	
	35	8272330_C/T	Intergenic	1.54E−06	OR	3.23	8272330	8596602	*SLC35B3*		
**Energy levels**	**15**	**41054337_G/A**	**Intergenic**	**6.55E** **−** **07**	**OR**	**2.26**	**39826935**	**41688715**	***PMCH***	*ANO4, SLC5A8, UTP20, ARL1, SPIC, MYBPC1, GNPTAB, DRAM1, NUP37, PARPBP, PMCH, IGF1,* and *PAH*	18.69 ± 7.08
**Separation related problems**	15	36773135_T/C	Intergenic	2.44E−06	OR	2.78	35227885	37839330	*NEDD1*	*VEZT, METAP2, USP44, NTN4, AMDHD1, HAL, LTA4H, ELK3, CDK17, CFAP54, NEDD1,* and *CCDC38*	32.21 ± 14.26
	33	22432039_G/A	Intergenic	1.10E−05	OR	2.35			*IGSF11*		
**Touch sensitivity**	**17**	**20787827_T/C**	**Intergenic**	**1.14E** **−** **05**	**OR**	**2.15**	**20787827**	**20810875**	***SLC35F6***	*SLC35F6*	25.49 ± 11.64
	**22**	**9094733_T/C**	**Intronic**	**4.14E** **−** **07**	**OR**	**2.02**	**7712048**	**11232729**	***VWA8***	*FAM216B, TNFSF11, AKAP11, DGKH, VWA8, NAA16, MTRF1, KBTBD6, WBP4, ELF1, SUGT1, CNMD, PCDH8, OLFM4, KBTBD7,* and *EPSTI1*	
	25	47544666_G/T	Intergenic	1.45E−05	OR	1.72			*ACKR3*		
**Trainability**	**25**	**49735848_C/T**	**Intergenic**	**2.11E** **−** **06**	**β**	**−** **0.10**	**49713759**	**49741131**	***ROMO1***		4.64 ± 3.12
	**6**	**65510825_C/T**	**Intergenic**	**5.32E** **−** **07**	**β**	**−** **0.08**			***ADGRL2***		
	**27**	**20876479_G/A**	**Intronic**	**1.68E** **−** **06**	**β**	**0.07**	**20876479**	**20435874**	***ITPR2***	*STK38L, TM7SF3, FGFR1OP2, INTS13, ITPR2,* and *MED21*	

Results from GWAS in which one or more variants surpassed the suggestive significance threshold including lead SNP, upper and lower bounds of regions of interest bounded by variants in high LD with the lead SNP (r^2^ ≥ 0.7). Also shown are the GWAS significance *P* value for the lead SNP, odds ratio (OR) for case:control studies or beta coefficient (β) for continuous traits, the proximal candidate gene (closest protein coding gene to lead SNP), positional candidate genes (additional protein coding genes which overlap the region of interest), and the SNP-heritability (h^2^ ± SE). DDA, Dog Directed Aggression; Chr, Chromosome. Genome-wide and suggestive significance was defined as *P* < 2.97 × 10^−6^ and *P* < 1 × 10^−5^, respectively. Genome-wide significant loci are shown in bold; suggestive loci are in regular font.

**Fig. 1.**
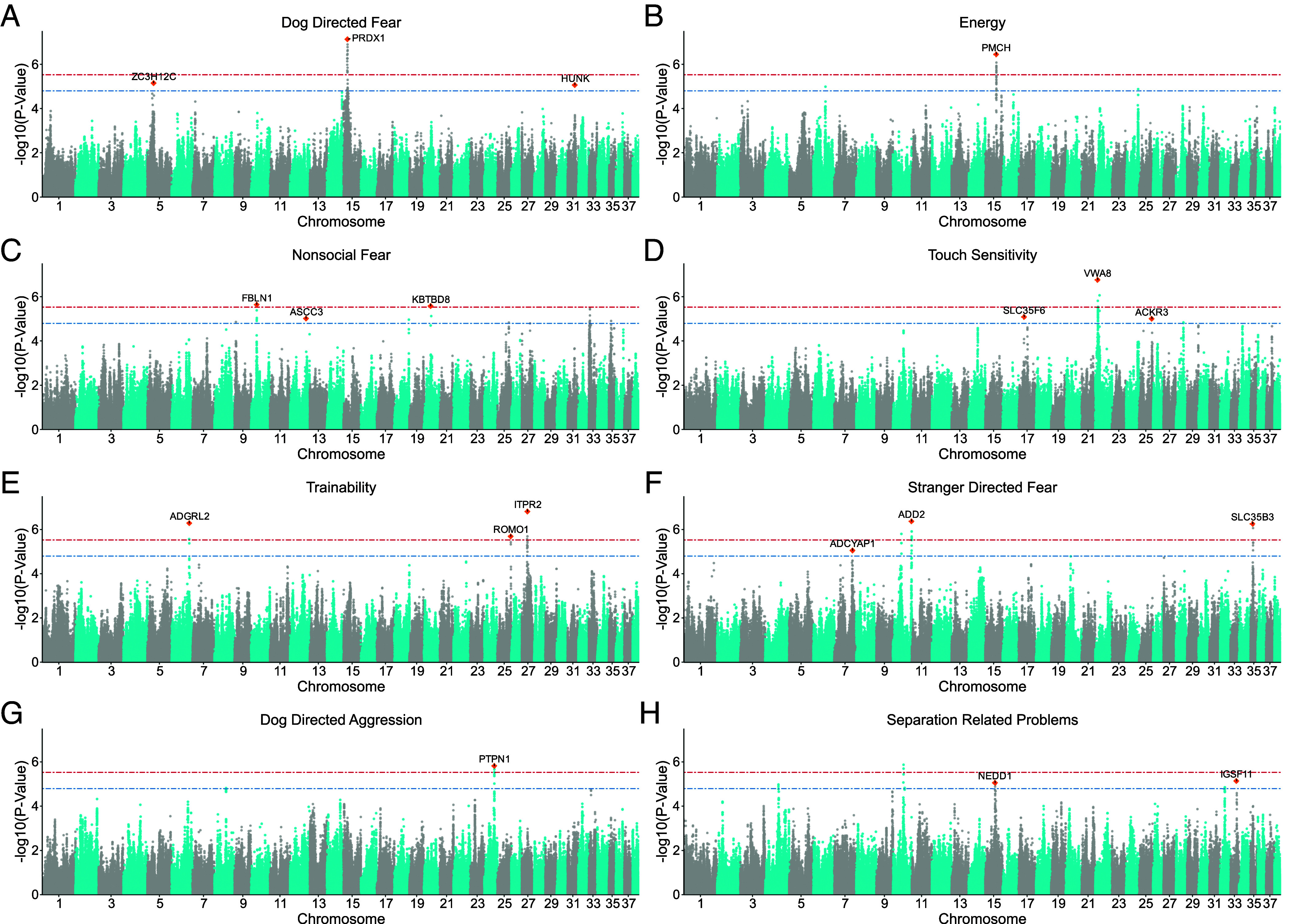
Manhattan plots of genome-wide association studies (GWAS) of behavioral traits in golden retrievers highlighting loci associated with C-BARQ factor scores. (*A*) dog-directed fear (n_case_ = 355, n_control_ = 128, covariate: neuter status), (*B*) score energy level (n_case_ = 248, n_control_ = 939, covariate: activity level), (*C*) non-social fear (n_case_ = 155, n_control_ = 281, covariates: age, neuter status, any disease, and activity level), (*D*) touch sensitivity (n_case_ = 171, n_control_ = 532, covariates: neuter status, and activity level), (*E*) trainability (n=1187, covariates: age, and activity level), (*F*) stranger-directed fear (n_case_ = 165, n_control_ = 630, covariates: age, and sex) (*G*) dog-directed aggression (n_case_ = 145, n_control_ = 455, covariates: sex, and activity level), and (*H*) separation-related problems (n_case_ = 128, n_control_ = 512, covariates: sex and age). The blue dashed line indicates suggestive significance, *P* < 1 × 10^−5^ and the red open dashed line indicates the Bonferroni-corrected significance threshold, *P* < 2.97 × 10^−6^. Lead SNP at each locus that exceeded the suggestive threshold are annotated with the proximal (closest) candidate gene. The loci where SNP surpass the significance threshold, but there are no annotations did not have any protein coding genes within the region of interest (plots *C*, *F*, and *H*). SNP, single nucleotide polymorphism; n_control_, number of control; n_case_, number of cases.

GWAS identified variants exceeding either the Bonferroni-corrected significance threshold (*P* = 2.97 × 10^−6^) or a conventionally used suggestive significance (*P* = 1 × 10^−5^) for 8 of 14 traits (Stranger directed fear, Dog directed fear, Non-social fear, Touch sensitivity, Separation-related problems, Trainability, Energy levels, and Dog-directed aggression). Each identified 1 to 3 independently associated loci (total 21, of which 12 surpassed the genome-wide significance threshold and 9 the suggestive significance threshold), containing 5-32 SNP (Fig. 1 and Dataset S1). Regions of interest (ROI) were defined as the region bounded by SNP in high LD with the lead variant at each locus (r^2^ ≥ 0.7), resulting in regions ranging from ~30 kb to 4.5 Mb. Where no other SNP were in LD with the lead variant, only the most proximal candidate gene was taken forward. Lead variants, regions of interest, and proximal and positional candidate genes are listed in Table 2.

**Table 2.** Canine genes identified in the C-BARQ GWAS had multiple significant PheWAS associations for human temperamental and cognitive traits

C-BARQ Factor	Canine genes	Human traits
**Dog-directed fear**	*ZC3H12C*	Educational attainment
	***PRDX1***	Educational attainment, Mania - Ever had period extreme irritability
	*HUNK*	Educational attainment, Worry too long after embarrassment, Ever smoked
**Non-social fear**	*ASCC3*	Mood swings, Miserableness, Irritability, Sensitivity/hurt feelings, Seen doctor (GP) for nerves, anxiety, tension or depression, Neuroticism score, Anxiety - Recent worrying too much about different things, Depression - Waking too early, Neuroticism, Depressive affect subcluster, Loneliness (MTAG), Depression, Well-being spectrum
**Stranger-directed fear**	***ADD2***	Depression - Professional informed about depression
**Dog-directed aggression**	***PTPN1***	Intelligence, Cognitive performance, Educational attainment, Major depressive disorder
**Trainability**	***ROMO1***	Fluid intelligence score, Cognitive performance, Depression - Recent feelings of depression
	***ADGRL2 (LPHN2)***	Intelligence, Irritability, Sensitivity/hurt feelings, Guilty feelings, Educational attainment
	***ITPR2***	Worry too long after embarrassment, Seen a psychiatrist for nerves, anxiety, tension or depression, Worry too long after embarrassment (WORR-EMB), Well-being spectrum
**Touch sensitivity**	*SLC35F6*	Risk taking, Sleep duration, Schizophrenia, Schizophrenia/Bipolar disorder
	***VWA8***	Educational attainment
**Separation-related problem**	*IGSF11*	Educational attainment

The proximal candidate genes identified at loci significantly associated with C-BARQ factor scores in the canine GWAS were interrogated for association with human temperamental and cognitive traits. Genes from loci reaching genome-wide significance in the canine GWAS are shown in bold, while those from suggestive loci are shown in regular font.

### Overlap With Previous Genetic Associations With Canine Behavior.

We investigated whether the loci we identified overlapped those in three previous studies testing genetic associations with C-BARQ behavioral traits and other behavioral and temperamental traits (14, 15, 71, 77–79). A proximal candidate gene at a suggestive locus for non-social fear (*KBTB8*, chromosome 20) was previously associated with separation-related problems in a multibreed population (14). Three genes which we identified within a genome-wide significant locus associated with dog-directed fear (*RAD54L*, *LURAP1,* and *CCDC17,* chromosome 15) were associated in that study with owner-directed aggression, energy level, and excitability respectively (14) and a gene we identified in a suggestive locus associated with Stranger-directed fear, *ENOSF1* (chromosome 7), was previously associated with energy level (14).

We identified a large genome-wide significant locus on chromosome 15 in the GWAS for Energy levels which contained 13 genes, with *PMCH* the most proximal gene to the lead SNP (lead SNP 15:41054337, region of interest 15:39826935-41688715). Associations with this locus or genes within it have been reported for several traits: Mahmoodi et al. (71) identified this as their lead association with multiple C-BARQ behavioral traits (dog-directed aggression, dog-directed fear, non-social fear, owner-directed aggression, separation-related behavior, touch sensitivity) and with a combined trait derived from those factors which they named “Dog Social Behaviors.” They separately reported an association between dog-directed fear and another gene on the same chromosome, insulin-like growth factor 1 (*IGF1*), variation in which is a major determinant of size in dogs (80–82). *IGF1* lies within the same locus in our study but that is hard to assess in the Mahmoodi data which did not report the LD structure across the region.

An IGF1 association was also identified in another study of fear-related traits (71, 79). Both studies relied on quantifying behavior in one cohort and studying associations using genomic data from dogs of the same breeds which had not been phenotyped. In studies where GWAS was performed using genetic data from directly phenotyped dogs, other positional candidate genes at this locus have been associated with curiosity (*ANO4*, in a cohort with diverse ancestry) (15), and poor performance in scent-detection Labrador retrievers (*DRAM1*) (77).

A common *IGF1* allele is a major determinant of size in dogs (83, 84). Therefore, we examined the relationship between energy level, body weight, and the lead SNP at this locus. Dogs carrying the alternate large size allele (G) had significantly lower energy levels (G at 15:41054337, OR 2.26 for being >90th centile of energy level, *P* = 6.55 × 10^−7^) and weighed more (β = 2.68 kg per copy, *P* = 9.75 × 10^−9^ in linear regression model) (*SI Appendix*, Fig. S4) compared to those with the ancestral small size allele (A). Since lower weight could be due to decreased skeletal size or having lower fat mass, we examined whether the variant was associated with body condition score (BCS), a well-validated measure of adiposity. There was no association between the lead SNP and BCS (β = −0.14, *P* = 0.08, linear regression). Additionally, there was no association between Energy level and either body weight (*P* = 0.40) or BCS (*P* = 0.81) in the cohort (*SI Appendix*, Fig. S4). A logistic regression model including the SNP, weight, and BCS confirmed that SNP genotype was a significant predictor of energy level group (OR = 2.20, *P* = 0.0014) but neither BCS nor body weight were significant predictors of energy level group (Body weight, OR = 0.9, *P* = 0.6; BCS, OR = 1.07, *P* = 0.6).

Several genes we identified for separation-related problems within a suggestive locus on chromosome 15 were previously associated with C-BARQ behavioral traits: *NTN4* with touch sensitivity in *a* study using breed-average genotypic information to map associated genes (14), and *NEDD1* and *CFAP54* with friendliness to people in a population of working Labrador retrievers and German shepherds (78). On chromosome 33, we identified *IGSF11* to be suggestively associated with separation-related problems; this gene had previously been associated with trainability in ref. 14. Finally, two genes at a genome-wide significant locus for Touch sensitivity on chromosome 22, *VWA8* and *ELF1*, were previously associated to owner-directed aggression and chasing, respectively (14).

### Identifying Possible Mechanistic Links.

For each of the candidate genes, we searched public databases to understand gene function and examined whether there is existing evidence to link the gene to behavior. Without follow-on genetic, epidemiological, and mechanistic work, those approaches remain merely hypothesis-generating, but findings are summarized in *SI Appendix*, Table S4 which shows that for many of the candidate genes investigated, there is a link to neurological development, function, or other pathways which could lead to the phenotypes investigated. We tested whether loci within each GWAS were enriched for genes involved in similar biological processes using MAGMA. No enrichment was identified.

### PheWAS Identifies Cross-Species Associations With Human Traits.

To evaluate the cross-species relevance of the canine C-BARQ GWAS results, we examined human orthologs of the most proximal candidate genes identified in our canine GWAS. We conducted a Phenome-Wide Association Study (PheWAS) using the Atlas of Complex Trait Genetics database (https://atlas.ctglab.nl) (85) which contains MAGMA gene-level associations for 19,436 protein-coding genes. We hypothesized that proximal candidate genes identified on the canine GWAS would also be associated with human temperament, psychiatric, and cognitive traits. This approach identified significant associations with human traits for 12 of the 18 proximal candidate genes investigated. Of these, 7 genes (*PTPN1*, *ADD2*, *ROMO1*, *ADGRL2*, *ITPR2*, *VWA8*, and *PRDX1*) were located at genome-wide significant loci (*P* < 2.97 × 10^−6^) while 5 genes (*ASCC3*, *ZC3H12C*, *HUNK*, *SLC35F6*, and *IGSF11*) were located at suggestive loci (*P* < 1 × 10^−5^) (See details in Table 2 and Dataset S2 and *SI Appendix,* Table S6). In contrast, when we queried for association with 196 unrelated traits including a variety of infectious, gastrointestinal, anatomic, neoplastic, and other phenotypes (Dataset S3), none of the genes were significantly associated.

## Discussion

We have conducted the largest within-breed GWAS of C-BARQ behavioral traits, studying golden retriever dogs and identifying multiple loci and candidate genes for association with canine behavioral and temperamental traits. Using a cross-species approach, we found that a large proportion of the positional candidate genes in dogs were also associated with comparable traits in humans, providing evidence of shared genetic and molecular mechanisms underlying complex behavioral traits in both species. The human associations may challenge common assumptions concerning the emotional underpinnings of certain behaviors in dogs.

For twelve of the eighteen positional candidate genes from the canine GWAS, we found significant human associations for psychiatric, temperamental, and cognitive traits. Some gene-trait overlaps showed compelling biological convergence, such as *ASCC3*, which was associated in dogs with non-social fear, and in humans with neuroticism, anxiety, sensitivity or hurt feelings, and multiple other related traits. The gene *ROMO1* was associated in dogs with trainability and in humans with cognitive performance and intelligence, and also with depression, irritability, sensitivity or hurt feelings. In other cases, the dog and human traits were even less well matched across the species but could be explained as likely to represent divergent phenotypes resulting from a common underlying temperamental predisposition, as for another gene associated with canine trainability, *ADGRL2*, which in humans is associated with guilty feelings, irritability, and sensitivity or hurt feelings. These results imply shared underlying neurobiological pathways and further suggest that, rather than mapping directly onto single behavioral/psychiatric traits, many of the associated genes may influence broader dimensions of emotional state or behavioral regulation. Such findings may provide testable hypotheses regarding the emotional basis of canine behaviors.

Dogs’ value as models of human behavior and related traits has previously been confirmed in studying compulsive behaviors, autism, and eating behavior (32, 34, 86, 87), so the cross-species associations were not unexpected. The genetic commonality we identified may be useful in two key ways. First, GWAS in humans have identified hundreds of loci associated with psychiatric traits, frequently with very small effect sizes. In contrast, the effect sizes/odds ratios we identified in dogs (Table 1) are larger. This may be because in dogs there is less cognitive elaboration of biologically driven variation (88), making it more straightforward to identify true associations. The canine data show that these genes can have a significant impact on behavior which means they should be considered as high-priority candidates for further study.

Second, the shared genetic basis of comparable traits across species may provide clinically relevant insight into the impact genetic variation can have on temperament, behavior, and mental health. From a canine perspective, many of the traits quantified by C-BARQ are considered undesirable by owners and clinical interventions are frequently focused on how dogs can be desensitized to environmental triggers. However, for the canine candidate genes identified for dog-directed fear, dog-directed aggression, and non-social fear, there was a striking recurrence of human associations with temperamental traits including neuroticism, worry, mood swings, irritability, sensitivity or hurt feelings, and mental health conditions including depression and anxiety. This may indicate that dogs carrying those risk alleles may have a genetically driven tendency to emotional states that make them vulnerable to developing these undesirable behaviors. This in turn may have clinical implications for how to manage such dogs.

In a similar fashion, recognizing that genes associated with “trainability” are linked to intelligence and other cognitive traits in people may have practical implications. For instance, many service dogs are trained by encouraging desired behaviors with positive reinforcers, including food, something that has been implicated in the high frequency of obesity-associated mutations in assistance dog populations (87, 89). While these methods are clearly effective, our data suggest that it could be favorable to develop tests which select and train service dogs based on a broader definition of intelligence. For instance, tests founded on cognitive models, rather than based on reinforcement contingency performance (90).

It was notable that in the human PheWAS, single genes were frequently significant for multiple related traits, corroborating the high genetic correlation for many psychiatric traits (4, 5) (Table 2). Similarly, when we compared the C-BARQ trait-associated genes from our study with past research in the field, there were several genes which had been identified before for similar, although not identical, traits. In each case, those overlaps were part of a group of traits between which the relationships were formalized in a recent study by Mahmoodi et al. (71) as “Dog Social Behaviors.” These authors used latent variable modeling to reduce the dimensionality of C-BARQ data into this derived variable which shared genetic associations with the component traits. Our human and canine data further reinforce that distinct behaviors (fear, aggression, energy, etc.) may reflect divergent endpoints of a genetically driven emotional state which, given the association with anxiety and depression in humans, one could hypothesize might be amenable to pharmacologic intervention.

Finally, by mapping these traits within a single breed, our data clarify associations found in multibreed studies that have previously been hard to interpret. A minority of genetic studies of canine behavior have used GWAS to map associations between paired individual genotypes and behavioral phenotypes, as we did (15, 77, 91). The majority have made use of publicly available genetic data combined with C-BARQ scores from a different population and have used statistical approaches that are most likely to identify genetic variants which are breed-defining, rather than those which segregate within breeds (15, 71, 79). Consequently, when they have identified genes known for their robust associations to breed-defining traits like size, it has been hard to interpret whether that was because of covariance of the behavioral and morphological traits and, even if the association is causal, whether it is due to confounding differences in environmental exposures related to the trait.

For instance, the small size allele at the locus containing Insulin-like growth factor-1 (*IGF1*) (81, 83) has repeatedly been associated with fear and aggression traits in studies using public genomic data (40, 92). *IGF1* is well recognized as a major determinant of size in dogs, related to its crucial role in growth and development (80–82). The behavioral associations with body size have previously been suggested to be spurious (15, 93, 94), perhaps because smaller dogs are treated differently or feel threatened more frequently and are, therefore, more likely to behave “badly.” This could generate a misleading association with behavior which is actually mediated by size and life experience. However, *IGF1* does also affect brain development in other species (80, 82) so it is also possible this is a true pleiotropic locus at which the *IGF1* allele affects both skeletal growth/size and brain development and behavior (69, 92). Studies including dogs of mixed ancestry have not been able to address this issue (15). However, our single breed GWAS for energy levels identified a large chromosome 15 locus including the *IGF1* gene. We showed golden retrievers carrying the allele which decreased Energy weighed significantly more [β = 2.68 kg per copy of the G allele (*P* = 9.75 × 10^−9^) *SI Appendix,* Fig. S4], consistent with findings in mixed ancestry studies (81, 83, 93, 95). Since golden retrievers are a large size dog breed, we considered this small difference in weight would be unlikely to impact on dogs’ life experience, which was supported by finding no association between Energy level and body weight across the cohort. We further considered whether lower energy levels might be because dogs were overweight, but there was no association between BCS and the variant at this locus, nor between energy level and BCS across the cohort. The data therefore suggest that this association with Energy level is real, either a genuine pleiotropic effect of the *IGF1* large size allele on both size (81, 83, 95) and brain development (80, 82) or due to a nearby linked variant affecting other genes.

It was notable that even GWAS for the canine behavioral traits with low heritability, particularly trainability (h^2^ = 4.6%, SE = 3.1) and dog-directed fear (h^2^ = 7.4%, SE = 12.4), detected multiple associated loci. This is likely to reflect the presence of a small number of variants of large effect which are more likely to emerge and become common in purebred canine populations (83, 96, 97). Such variants may be detected using GWAS even while SNP-based heritability remains modest (98, 99). Importantly, the wide SE for several traits, especially for Dog-directed fear, means that the exact magnitude of heritability remains uncertain. These high-SE likely arise from the sparsity of nonzero behavioral scores (i.e. most dogs did not display the behavior) and from the modest sample size for GREML-LDMS modeling (100–104). However, it is important to note that the large SE does not necessarily bias the point estimate but instead widens the CI (103). Despite these limitations, the relevance of several implicated loci is bolstered by the fact that proximal candidate genes at these loci were associated with related human traits.

Investigating the genetic basis of these traits in a single breed capitalizes on the genetic architecture of purebred dogs which enhances power to detect associations with only modest sample sizes, an approach proven previously for complex behaviors in canine breed populations (31, 105). Nonetheless, this analysis will by no means have identified all the genetic variants responsible for determining these traits in golden retrievers. Further, it is important to remember that the SNP associations identified in this breed may be absent in other breeds in which different LD structure means the same SNP may not be linked to the causative variants, or the causative variants are absent (9, 14, 15).

It is important to note that, as with any study involving privately owned dogs, our subjects were inevitably exposed to a range of environmental risk factors such as differences in home environments, training routines, nutritional regimens, and exercise levels that could influence behavioral expression. Similarly, owner-reported measures to quantify behavior are inevitably vulnerable to subjectivity in owners’ assessment of their dogs’ actions (73). To mitigate, efforts were made to avoid bias by not including more than one dog per household, incorporating a GRM into the LMM to account in some part for not only genetic but some early-life environmental effects (e.g., litter conditions, breeder practices). Although these confounding factors are likely to have reduced the study’s power to identify true genetic associations, there is no reason to believe they would have introduced false positive associations.

The C-BARQ scores we report from the GRLS dogs are like those reported for golden retrievers previously, with comparable score distributions and associations with factors such as sex, neuter status, and age (15, 23–31). Although we did not conduct formal statistical comparisons against other breed datasets, these observations are consistent with published findings indicating that golden retrievers often exhibit higher trainability and lower aggression and dog rivalry scores compared to many other breeds reported previously (15, 24–29, 32). Importantly, we selected only dogs aged 3 to 7 y old, meaning that the regression models generated to select GWAS covariates are only applicable to this population and should not be generalized to golden retrievers in general. For instance, by selecting only middle-aged dogs we did not find an association between age and excitability, despite puppies previously having been shown repeatedly to be more excitable (106, 107).

In conclusion, we have performed the largest single-breed GWAS for C-BARQ behavioral traits in dogs, identifying significant genetic associations and providing insight into the genetic architecture of behavioral traits in the species. Using a hypothesis-driven human comparative PheWAS, we identified multiple genes which are associated with temperament, mental health, and cognitive abilities across both species. These provide orthogonal evidence of their influence on behavior from a second species and therefore highlighting them as worthy of future mechanistic study.

## Methods

The methods used in the study are outlined here in brief, but there is more information on the details of the analysis and rationale for the approach adopted in the *SI Appendix*, *Supplementary Text Materials and Method*.

### Study Population and C-BARQ Phenotypes.

This study used data from the Golden Retriever Lifetime Study (GRLS). The overall study design, including the details of the sampling method, and criteria for inclusion and exclusion have been previously documented in detail (41, 108). In brief, however, the GRLS is a prospective cohort study designed to evaluate nutritional, environmental, lifestyle, and genetic risk factors for the development of cancer and other diseases in golden retrievers. Between 2012 and 2015, a total of 3,044 purebred golden retrievers aged between 6 mo 2 y old and resident in the contiguous United States were enrolled. Each year pet owners provided consent and completed a detailed survey about their demographics, lifestyle, and health, including C-BARQ. By mid-2021, there were 2,251 active subjects in the GRLS, (352 dogs deceased, 441 lost to follow-up, retention rate of 86%). The retention strategies and attrition have also been described by Labadie et al. (109). All canine samples and data were collected under a study protocol that was approved by Morris Animal Foundation’s Animal Welfare Advisory Board as part of the GRLS (41, 42). All data are available via the GRLS Data Commons portal (https://www.morrisanimalfoundation.org/data-commons).

Behavioral traits were assessed using the Canine Behavioral Assessment and Research Questionnaire (C-BARQ) (43). Only dogs with complete C-BARQ responses from a single occasion were included and, if owners had responded repeatedly, we selected data from a single time point at random. We only included data from dogs aged 3 to 7 y to minimize variation due to developmental changes in puppyhood and old age-related decline, and because dogs of this age have typically established consistent routines and social behaviors, making owner reported assessment more reliable. The C-BARQ questionnaire includes 100 items, of which 78 have previously been grouped into 14 behavioral traits, each of which was scored as defined previously (questionnaire items and scoring system in *SI Appendix*, Table S1 and *Supplementary Text Materials and Method*). The scores for the 14 C-BARQ behavioral items were the focus of our genetic study

### Canine GWAS.

DNA was extracted from EDTA whole blood samples and stored at −80 °C (42) before array genotyping using ThermoFisher Scientific’s Axiom™ Canine Genotyping Array Sets A and B to call approximately 1.1 million markers, with positions reported on CanFam3.1 reference assembly (41, 42). PLINK v1.9 software (110) was used to filter out markers with >5% genotype calls missing, which deviated from Hardy–Weinberg equilibrium (*P* < 1e−6), or with minor allele frequency <5%, and individuals with >5% genotype calls missing. Identity by Descent estimates were used to remove strongly related dogs where PI_HAT > 0.25. We tested for stratification and outliers by creating a centered relatedness matrix using the Genome-wide Efficient Mixed Model Analysis (GEMMA) Software v0.98.1 (75) which was transformed into distance matrices using the tidyverse package in R.4.2.2 (111) and visualized them on multidimensional scaling plots. The final dataset included 468,649 markers (call rate of 99%) from a maximum of 1,187 dogs.

For each C-BARQ trait, we first examined the distribution of scores within the population to determine the appropriate analytical approach. Traits with approximately normal distributions were analyzed as continuous variables using linear regression. For traits with heavily skewed distributions and zero inflation, data transformation did not normalize the distributions to meet the assumptions for GWAS, therefore a case–control approach was employed (*SI Appendix*, *Supplementary Text Materials and Method*). Regression modeling was used to determine population-specific covariates relevant to each C-BARQ trait in the GWAS.

Heritability was estimated using the genome-based restricted maximum likelihood (GREML) approach, implemented through GCTA software (v.1.93.2) (76) specifically employing the GREML-LDMS approach to adjust for the influence of LD and minor allele frequency (MAF) on the estimated SNP heritability. GWAS was conducted using a univariate linear mixed model in GEMMA v0.98, with covariates included determined by the regression modeling (*SI Appendix*, Table S5) plus a centered relatedness matrix, with significance determined by the likelihood ratio test (LRT). Genome-wide significance thresholds were set using Bonferroni correction (*P* = 0.05/number of independent variants, yielding a threshold of 2.97 × 10^−6^); a suggestive threshold of 1 × 10^−5^ was also used.

For each locus of interest, we defined a wider region of interest as that bounded by SNP in high LD (r^2^ ≥ 0.7). The gene-based and gene-set enrichment analyses were performed using MAGMA *v1.10* on GWAS summary statistics for 14 C-BARQ behavioral traits. We investigated whether either proximal or positional candidate genes identified in our GWAS had been reported in previous GWAS for C-BARQ and other canine behavior related traits (9, 10, 14, 18, 40, 77, 78, 92, 112). An Ovid MEDLINE® (Embase <1,996 to 2,024 wk 44>) (113) advanced search strategy was employed, targeting journal articles through keyword mapping to focused subject heading in October 2024.

### Human Comparative Analyses.

To evaluate cross-species relevance, we examined human orthologs of candidate genes identified in our canine GWAS. We conducted a Phenome-Wide Association Study (PheWAS) using the Atlas of Complex Trait Genetics (ATG) database (https://atlas.ctglab.nl) (85), focusing on the genes most proximal to the top SNP at each suggestive or significant C-BARQ locus. The ATG database provides precalculated MAGMA gene-level p-value. MAGMA was performed using 19,436 protein coding genes obtained from biomaRt after assigning SNPs to genes using 1 kb window on either side. The LD reference panel was based on either the 1,000 Genomes Project or UK Biobank, depending on the GWAS. Analysis was restricted to GWASs with N ≥ 45,000. Given that we were testing predefined genes identified from the canine GWAS across multiple traits, our primary multiple testing burden was at the trait level. We applied a Bonferroni-corrected significance threshold of *P* < 2.64 × 10^−4^ (0.05/190) for psychiatric, temperamental, and cognitive traits (190 traits in total see Dataset S2). To determine whether the associations we identified were greater than expected by chance, we tested the same 18 genes for association with 196 unrelated traits, applying a Bonferroni-corrected significance threshold of *P* < 2.55 × 10^−4^ (0.05/196, traits listed in Dataset S3).

## Supplementary Material

Appendix 01 (PDF)

Dataset S01 (XLSX)

Dataset S02 (XLSX)

Dataset S03 (XLSX)

## Data Availability

All study data are included in the article and/or supporting information.
