# Supplementary material for: GWAS for behavioral traits in golden retrievers identifies genes implicated in human temperament, mental health, and cognition
Source: Proc Natl Acad Sci U S A. 2025 Nov 24;122(48):e2421757122. doi: 10.1073/pnas.2421757122 (PMC12684936; doi:10.1073/pnas.2421757122)
Supplement: Supplementary file 1 — Appendix 01 (PDF) [file pnas.2421757122.sapp.pdf]

Supporting Information for

GWAS for behavioral traits in golden retrievers identifies genes implicated in human temperament, mental health, and cognition.

Enoch Alex[1], Paul Gennotte[1], Anna Morros Nuevo[1], Yunzhu Yu[1], Benjamin Keep[1,2], Daniel Mills[3], Varun Warrier[4,5], Eleanor Raffan[1]*.

[1] Dept. Physiology, Development and Neuroscience, University of Cambridge, UK.

[2] School of Biological and Behavioural Sciences, Queen Mary University of London, UK.

[3] Dept. Life Sciences, University of Lincoln, UK.

[4] Dept. of Psychiatry, University of Cambridge, UK

[5] Dept. of Psychology, University of Cambridge, UK

***Corresponding Author:** Eleanor Raffan

Email: er311@cam.ac.uk

This PDF file includes:

- Supporting text Materials and Methods
- Figures S1 to S4
- Tables S1 to S6
- Legends for Datasets S1 to S3
- SI References

Other supporting materials for this manuscript include the following:

- Datasets S1 to S3

Supporting Text

Supplementary Materials and Methods

Phenotype Selection and Regression modelling for C-BARQ factor scores.

For each C-BARQ behavioral trait, we first examined the distribution of scores within the population to determine the appropriate analytical approach. Traits with approximately normal distributions were analyzed as continuous variables using linear regression. For traits with heavily skewed distributions and zero inflation, we adopted a case-control approach. This is because using deterministic or monotonic transformations on such zero-inflated traits without introducing randomness, will inherently preserve order and exacerbate the clustering at zero. To define cases and controls for these traits, we identified individuals with the most extreme behaviors as cases, using the 90th percentile as a cutoff for most traits (1–3).

For the purpose of downstream GWAS analysis, controls were drawn randomly from dogs scoring 0 for the respective traits to achieve a 1:4 case-control ratio. The choice of this ratio stems from the challenges associated with dealing with extreme case-control imbalance in GWAS which results in high Type I error rates, biased estimates, and reduced power to capture the real association due to violated asymptotic assumptions in regression models (4–6). While more recent tools like Scalable and Accurate Implementation of Generalized mixed mode (SAIGE) (4) and BOLT-LMM (7) have been developed to address such imbalances, they require large sample sizes (typically exceeding 10,000 for SAIGE and 5,000 for BOLT-LMM). Given that our dataset contains fewer than 1,400 samples, these methods were not feasible for our analysis. Dai et al. (6) also reported that while SAIGE and BOLT-LMM are powerful for large-scale data, their performance is limited in smaller datasets due to the challenges of sample size and variance estimation. To address these issues, we employed a random under-sampling method selecting a 1:4 ratio as a practical compromise. This reduces the extreme disparity between cases and controls while ensuring adequate sample representation (4, 6).

For Energy level, scores were approximately normally distributed but we hypothesized major gene effects would be more powerfully identified by comparing the most energetic dogs with controls drawn from the rest of the population, so we adopted a case-control approach, defining individuals in the top 10% of the distribution as cases with controls drawn from the remaining 90% of the distribution. Table S2 includes the thresholds used for each case:control study, the number of dogs in each group and compares the summary statistics for each variable included in the modelling.

Regression modelling was used to determine population-specific covariates relevant to each CBARQ behavioral trait in the GWAS. We employed a stepwise model selection method based on Akaike's Information Criterion (AIC), which optimizes model fit while minimizing overfitting by penalizing unnecessary parameters. Each initial model included age, sex, neutered status, interactions of sex with neutered status, dog purpose (pet vs. service dog), activity level, and disease status. Covariates were retained only if they significantly reduced AIC values, resulting in a minimal model specific to each trait. Variables retained in the minimal models with significant effects were subsequently used as covariates in the GWAS analyses. Detailed information on the covariate selection process, including AIC values and p-values for each trait, is provided in *SI Appendix,* Table S5.

Heritability Analysis

Heritability was estimated using the genome-based restricted maximum likelihood (GREML) approach, implemented through GCTA software (v.1.93.2) (8) specifically employing the GREML-LDMS approach to adjust for the influence of linkage disequilibrium (LD) and minor allele frequency (MAF) on the estimated SNP heritability. The analysis was conducted in several steps. First, LD scores were calculated for segments across the genome, using an LD window size of 200 kilobases (kb) with an overlap of 100 kb between neighboring regions. Next, the stratified SNPs, grouped based on their segment-specific LD scores, were processed in R (v.4.2.2). Multiple genetic relatedness matrices (GRMs) were then generated from the stratified SNPs. These GRMs, along with relevant covariates, were integrated into the restricted maximum likelihood (REML) analysis to derive heritability estimates (*SI Appendix*, Table S3).

Calculating the Genomic Inflation Factor (λ)

The genomic inflation factor (lambda, λ) was calculated with the function P lambda in the R package QCEWAS (9).

Generating Visualization Plots

Visualization of Manhattan and Quantile-Quantile (QQ) plots was performed in R 4.2.2 using the qqman package.

Transforming Effect Size Estimates

To interpret the meaning of the linear mixed model (LMM) coefficients in terms of odds ratios (ORs), we transformed LMM-derived effect sizes for binary traits as previously described (10).

Gene Set Enrichment Analysis and Comparison with other canine behavior GWAS

The gene-based and gene-set enrichment analyses were performed using MAGMA *v1.10* on GWAS summary statistics for 14 CBARQ behavioral traits. In the gene-based analysis, MAGMA mapped SNPs to 19,056 protein-coding genes, computing gene-level p-values using the SNP-wise mean model. The model accounts for the number of SNPs within each gene, the gene size, and LD among SNPs within the gene region. To assess whether genes within a predefined gene set showed significant associations with CBARQ behavioral traits compared to genes outside the set, the competitive testing framework for the gene-set enrichment analysis in MAGMA was used. Gene sets analyzed included 6,533 Gene Ontology (GO) terms and 359 KEGG canonical pathways, curated from the R/Bioconductor package *org.Cf.eg.db* (*v. 3.19.1*) for GO terms and the *clusterProfiler* package (*v. 4.12.6*) for KEGG pathways.

Comparison with Previous CBARQ GWAS Studies

We investigated whether either proximal or positional candidate genes identified in our GWAS had been reported in previous GWAS for C-BARQ and other canine behavior related traits. An Ovid MEDLINE® (Embase <1996 to 2024 Week 44>) advanced search strategy was employed (11), targeting journal articles through keyword mapping to focused subject heading on October 2024.

Keywords included '*genome-wide association study/' AND (dog* OR canine) AND ('canine behavior assessment questionnaire' OR 'c-barq'), returning 5 studies. One of them contained GWAS of C-BARQ behavioral traits in multi breed populations (12), and the remaining used a similar or derived set of questions to assess behavior in one (13–15) or two breed (16) samples. Further relevant studies were identified that utilized similar methodologies with a population of German shepherd (17, 18) and Labrador retriever dogs (19), a study that mapped fear and aggression traits across multiple breeds (20), a study which mapped the genetic basis of C-BARQ behavioral traits in many different breeds (21) and another GWAS for performance in scent-detection Labrador retrievers (14).

Human Comparative Analysis

To evaluate cross-species relevance, we examined human orthologs of candidate genes identified in our canine GWAS. We conducted a Phenome-Wide Association Study (PheWAS) using the Atlas of Complex Trait Genetics (ATG) database (https://atlas.ctglab.nl) (73). We selected the ATG database because it is a well-organized repository of GWAS data available and is particularly well suited for psychiatric and cognitive traits that are underrepresented in other similar databases. Notably, the ATG team has performed GWAS for many of those traits from UK Biobank data. Our comparative analysis included 190 human behavioral, psychiatric, and cognition related traits. Broadly, this comprises 112 temperament-related and 78 cognition-related traits.

The ATG database provides pre-calculated MAGMA gene-level p-values, which were computed using 19,436 protein-coding genes obtained from biomaRt after assigning SNPs to genes using a 1 kb window on either side. The linkage disequilibrium (LD) reference panel was based on either the 1000 Genomes Project or UK Biobank, depending on the GWAS under consideration. Analyses were further restricted to GWAS studies with sample sizes of N ≥ 45,000. Significant associations were determined using Bonferroni-corrected $p < 2.64\times10^{-4}$ for temperament and cognition related traits (190 traits in total).

Supplementary Figures

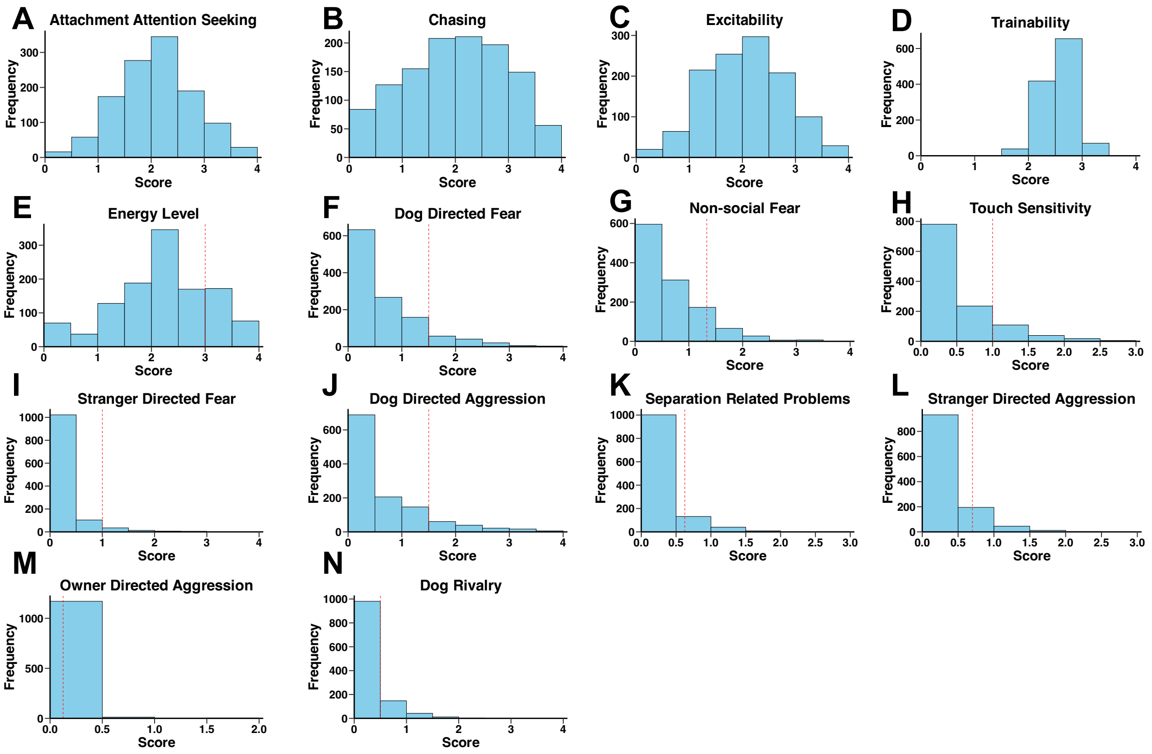

Fig. S1. Frequency distribution of C-BARQ behavioral traits.

For each trait, histograms are used to show the distribution of scores in the entire population of dogs. Normally distributed traits are analyzed as a continuous variable in the GWAS A) Attachment Attention Seeking, B) Chasing, C) Excitability, D) Trainability. The GWAS was analyzed on a case:control basis with cases defined as dogs scoring ≥90th percentile, or ≥95th percentile for Stranger-directed fear (cut-off shown with red dashed lines). Controls were sampled at random to achieve a 1:4 case:control ratio from dogs scoring 0 for the trait or, for Energy Level from all dogs in $<90^{th}$ percentile. Those traits are E) Energy Level, F) Dog Directed Fear, G) Non-social Fear, H) Touch Sensitivity, I) Stranger Directed Fear, J) Dog Directed Aggression, K) Separation Related Problem, L) Stranger Directed Aggression, M) Owner Directed Aggression, and N) Dog Rivalry.

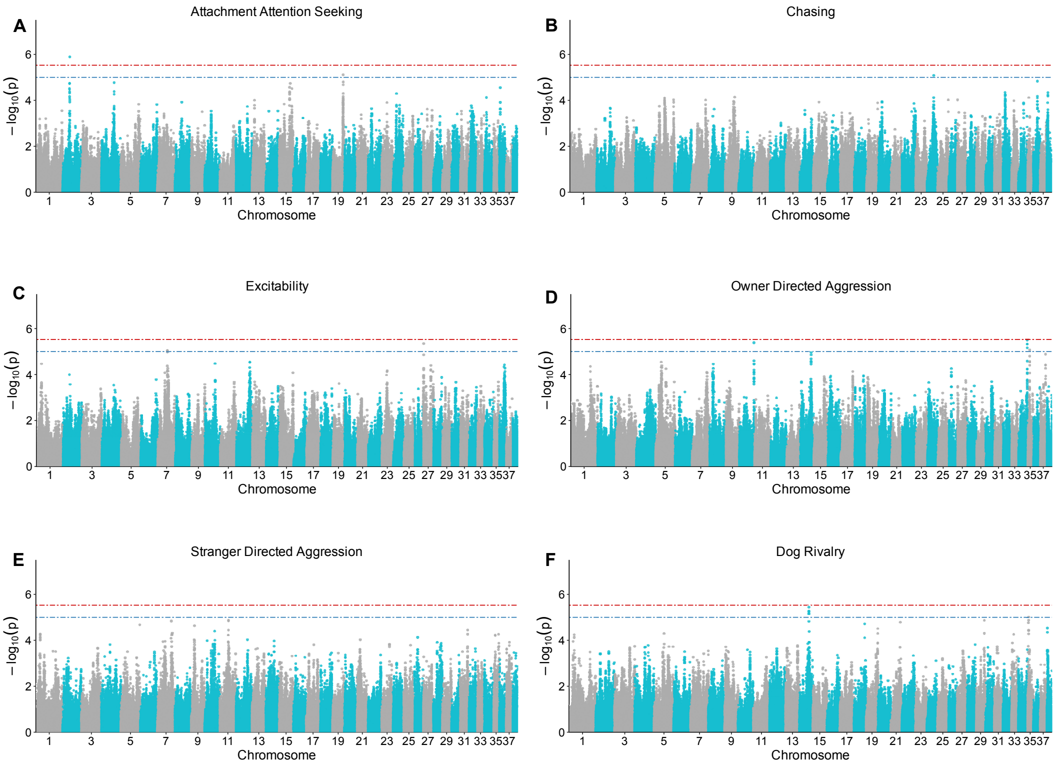

Fig. S2. Manhattan plots of genome-wide association studies of C-BARQ behavioral traits in golden retrievers highlighting associated loci. (A) Attachment Attention Seeking (n=1187), (B) Chasing (n=1187), (C) Excitability (n=1187), (D) Owner Directed Aggression (n_{case} = 122, $n_{control}$ = 480), (E) Stranger Directed Aggression (n_{case} = 141, $n_{control}$ = 507), and (F) Dog Rivalry (n_{case} = 206, $n_{control}$ = 824). The blue dashed line indicates suggestive significance, $p < 1\times10^{-5}$, and the red dashed line indicates the Bonferroni-corrected significance threshold, $p < 2.967\times10^{-6}$.

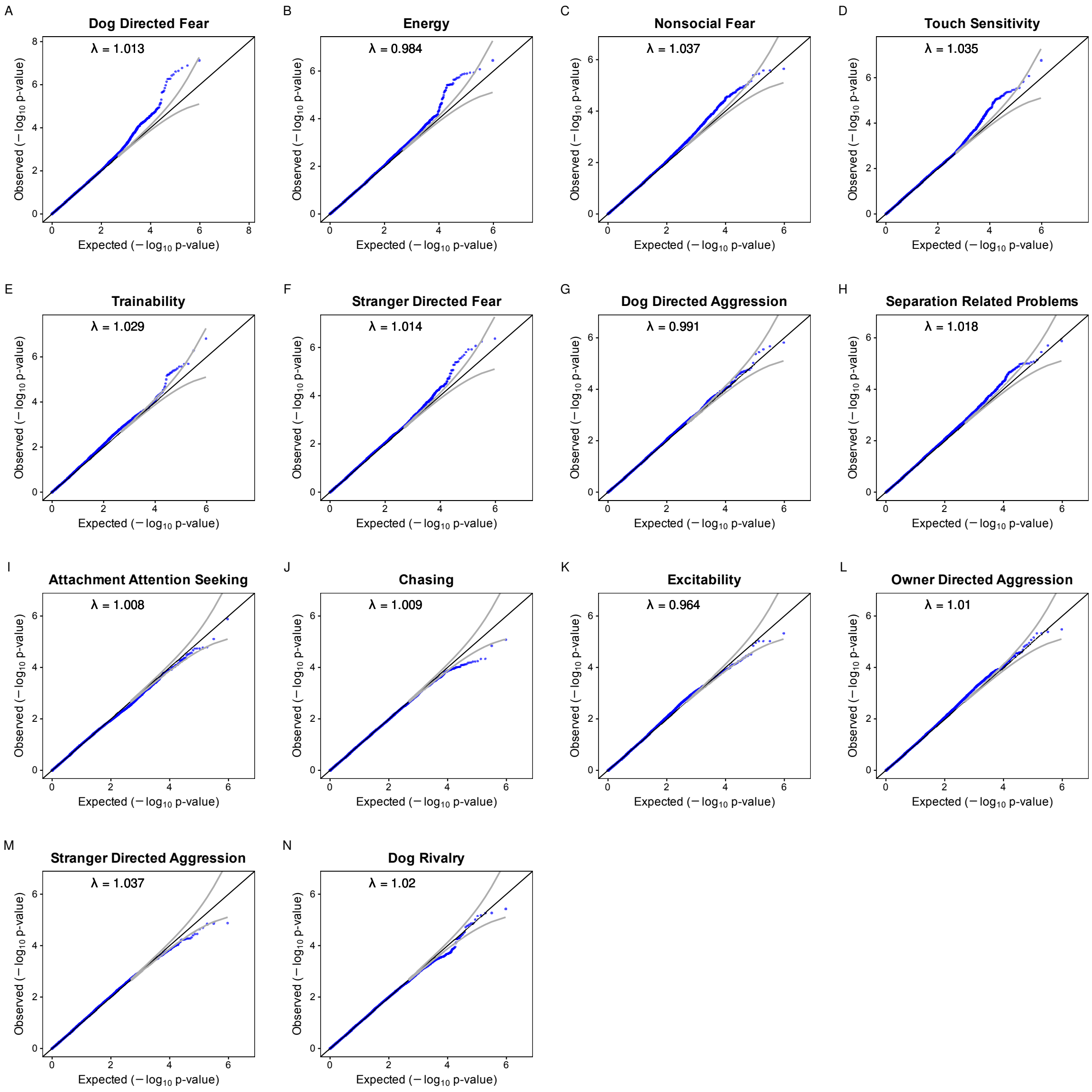

Fig. S3. Observed – Expected p value (Q-Q) plots for each C-BARQ behavioral trait with significant GWAS results. The diagonal line in each plot represents the expected distribution of p-values in the GWAS under the null hypothesis. The shaded area is the 95% confidence interval. Genomic Inflation Factor (λ) is overlain for each trait. (A) Dog-directed fear, (B) Energy level, (C) Non-social fear, (D) Touch sensitivity, (E) Trainability, (F) Stranger-directed fear, (G) Dog-directed aggression, and (H) Separation related problems, (I) Attachment Attention Seeking, (J) Chasing, (K) Excitability, (L) Owner Directed Aggression, (M) Stranger Directed Aggression, and (N) Dog Rivalry.

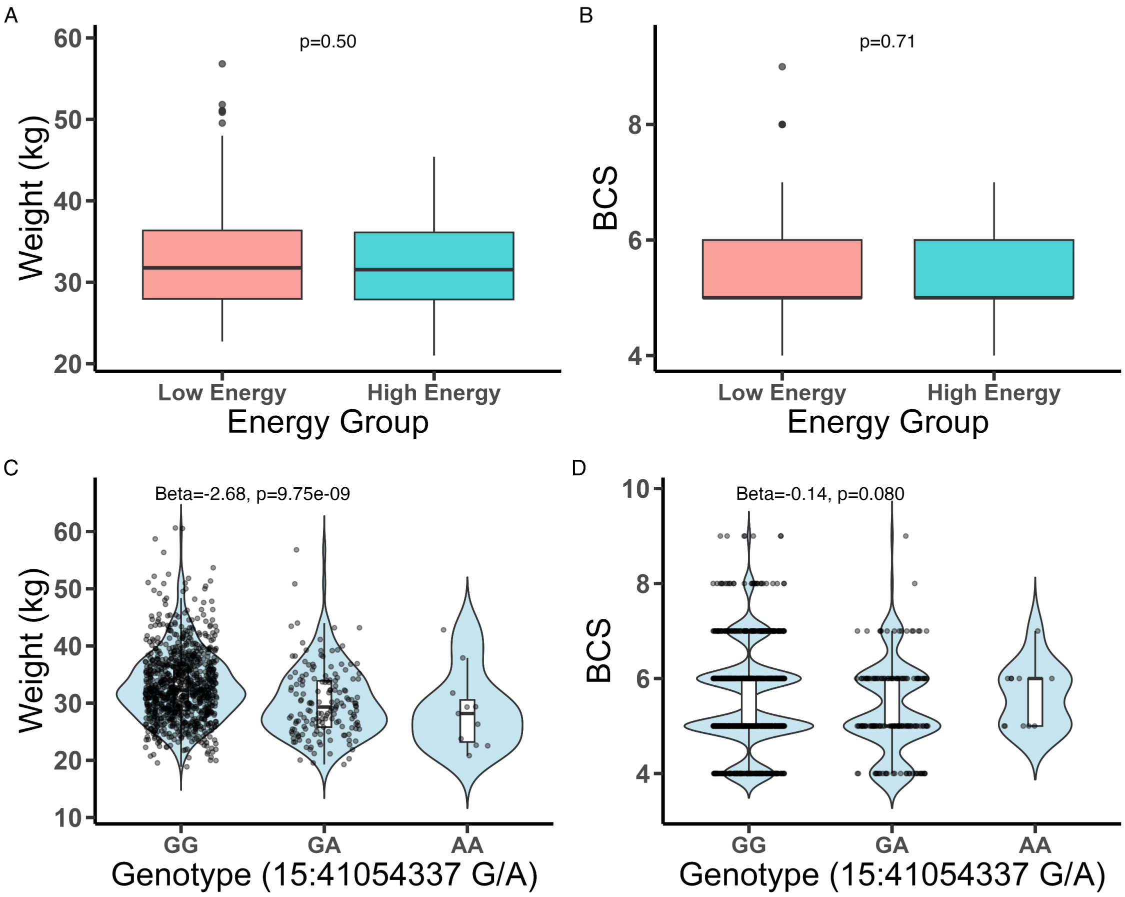

Fig. S4. Association of energy phenotype and chromosome 15 top SNP genotype with body weight and body condition score.
(A) Comparison of body weight between dogs categorized as Low Energy and High Energy. No significant difference was observed (Wilcoxon rank-sum test; p = 0.50). Boxplots show medians, interquartile ranges (IQR), and whiskers extending to 1.5 x IQR; individual data points are jittered.
(B) Comparison of body condition scores (BCS) between Low Energy and High Energy groups. No significant difference detected (Wilcoxon rank-sum test; p = 0.71).
(C) Violin plot showing the relationship between body weight and genotype at SNP 15:41054337_G/A within the chromosome 15 locus containing IGF1. Each additional copy of the G allele was associated with an increase of 2.68 kg in weight (linear regression; $\beta = 2.68$ kg, $p = 9.75 \times 10^{-9}$). Individual dogs are represented as jittered points.
(D) Effect of the same IGF1-region genotype on body condition scores. A slight, non-significant increase in BCS per G allele copy was observed (linear regression; $\beta = 0.14$, $p = 0.08$).

Supplementary Tables

Table S1. The C-BARQ scoring system.

The Canine Behavioral Assessment and Research Questionnaire (CBARQ) (22) provides quantitative scores for 14 different categories of behavior (C-BARQ behavior traits). The questionnaire is provided to the respondent divided into 7 Sections as outlined below where a five-point Likert scale for frequency or severity scores is used alongside a 'not observed/not applicable' option. Further details of C-BARQ data collection in the Golden Retriever Lifetime Study can be found here:
https://datacommons.morrisanimalfoundation.org/artisanal_dataset/121

Section 1 – Training and obedience.
Some dogs are more obedient and trainable than others. By clicking on the appropriate choices, please indicate how trainable or obedient your dog has been in each of the following situations in the recent past:
Where 'Never' = 0, 'Seldom' = 1, 'Sometimes' = 2, 'Usually' = 3 and 'Always' = 4

1. Is off leash, returns when called.
2. Obeys "sit" command immediately.
3. Obeys "stay" command immediately.
4. Seems to attend/listen closely to everything you say or do.
5. Is slow to respond to correction or punishment, "thick-skinned".
6. Is slow to learn new tricks or tasks.
7. Is easily distracted by interesting sights, sounds or smells.
8. Will "fetch" or attempt to fetch sticks, balls or objects.

Section 2 – Aggression
Some dogs display aggressive behavior from time to time. Typical signs of moderate aggression in dogs include barking, growling and baring teeth. More serious aggression generally includes snapping, lunging, biting, or attempting to bite. By clicking on the following scales, please indicate your own dog's recent tendency to display aggressive behavior in each of the following context:
Five-point Likert scale is used from 'No aggression' = 0 to 'Serious aggression' = 4

9. When verbally corrected or punished (scolded, shouted at, etc.) by owner or a household member.
10. When approached directly by an unfamiliar **adult** while being walked/exercised on a leash.
11. When approached directly by an unfamiliar **child** while being walked/exercised on a leash.
12. Towards unfamiliar people approaching the dog while s/he is in your car (at the gas station, for example).
13. When toys, bones or other objects are taken away by a household member.
14. When bathed or groomed by a household member.
15. When an unfamiliar person approaches you or another member of your family at home.
16. When unfamiliar people approach you or another member of your family away from your home.
17. When approached directly by a household member while s/he is eating.
18. When mailmen or other delivery workers approach your home.
19. When his/her food is taken away by a household member.
20. When strangers walk past your home while your dog is outside in the yard.
21. When an unfamiliar person tries to touch or pet the dog.
22. When joggers, cyclists, rollerbladers or skateboarders pass your home while your dog is outside or in the yard.
23. When approached directly by an unfamiliar **male** dog being walked/exercised on a leash.
24. When approached directly by an unfamiliar **female** dog being walked/exercised on a leash.
25. When stared at directly by a member of the household.
26. Toward unfamiliar dogs visiting your home.
27. Toward cats, squirrels or other animals entering your yard.
28. Toward unfamiliar people visiting your home.

29. When barked, growled, or lunged at by another (unfamiliar) dog.
30. When stepped over by a member of the household.
31. When you or a household member retrieves food or objects stolen by the dog.
32. Towards another (familiar) dog in your household.
33. When approached at a favorite resting/sleeping place by another (familiar) dog.
34. When approached while eating by another (familiar) household dog.
35. When approached while playing with/chewing a favorite toy, bone, object, etc. by another (familiar) household dog.

Are there any other situations in which your dog is sometimes aggressive? If so, please describe briefly.

Section 3 – Fear and anxiety

Dogs sometimes show signs of anxiety or fear when exposed to particular sounds, objects, people or situations. Typical signs of mild to moderate fear include avoiding eye contact, avoiding feared object, crouching or cringing with tail lowered or tucked between the legs, whimpering and whining, freezing, and shaking and trembling. Extreme fear is characterized by exaggerated cowering, and/or vigorous attempts to escape, retreat or hide from the feared object, person or situation. By clicking on the following scales, please indicate your own dog's recent tendency to display fearful behavior in each of the following contexts:

Five-point Likert scale is used from 'No fear or anxiety= 0 to 'Extreme fear' = 4

36. When approached directly by an unfamiliar **adult** while away from your home.
37. When approached directly by an unfamiliar **child** while away from your home.
38. In response to sudden or loud noises (e.g. vacuum cleaner, car backfire, road drills, objects being dropped, etc.).
39. When unfamiliar people visit your home.
40. When an unfamiliar person tries to touch or pet the dog.
41. In heavy traffic.
42. In response to strange or unfamiliar objects on or near the sidewalk *e.g. plastic trash bags, leaves, litter, flags flapping, etc.).
43. When examined/treated by a veterinarian.
44. During thunderstorms, firework displays, or similar events.
45. When approached directly by an unfamiliar dog of the same or larger size.
46. When approached directly by an unfamiliar dog of smaller size.
47. When first exposed to unfamiliar situations (e.g. first car trip, first time in elevator, first visit to veterinarian, etc.).
48. In response to wind or wind-blown objects.
49. When having nails clipped by a household member.
50. When groomed or bathed by a household member.
51. When having his/her feet toweled by a member of the household.
52. When unfamiliar dogs visit your home.
53. When barked, growled, or lunged at by an unfamiliar dog.

Section 4 – Separation-related problems

Some dogs show signs of anxiety or abnormal behavior when left alone, even for relatively short periods of time. Thinking back over the recent past, how often has your dog shown each of the following signs of separation-related behavior when left, or about to be left, on its own:

Where 'Never' = 0, 'Seldom' = 1, 'Sometimes' = 2, 'Usually' = 3 and 'Always' = 4

54. Shaking, shivering, or trembling
55. Excessive salivation.
56. Restlessness, agitation, or pacing.
57. Whining.
58. Barking.
59. Howling.
60. Chewing or scratching doors, floor, windows, curtains, etc.
61. Loss of appetite.

Are there any other situations in which your dog is fearful or anxious? If so, please describe briefly:

Section 5 – Excitability

Some dogs show relatively little reaction to sudden or potentially exciting events and disturbances in their environment, while others become highly excited at the slightest novelty. Signs of mild to moderate excitability include increased alertness, movement towards the source of novelty, and brief episodes of barking. Extreme excitability is characterized by a

general tendency to overreact. The excitable dog barks or yelps hysterically at the slightest disturbance, rushes towards and around any source of excitement, and is difficult to calm down. By clicking on the following scales, please indicate your own dog's recent tendency to become excitable in each of the following contexts:

Five-point Likert scale is used from 'Calm= 0 to 'Serious excitable= 4

62. When you or other members of the household come home after a brief absence.
63. When playing with you or other members of your household.
64. When the doorbell rings.
65. Just before being taken for a walk.
66. Just before being taken on a car trip.
67. When visitors arrive at your home.

Are there any other situations in which your dog sometimes becomes over-excited? If so, please describe briefly.

Section 6 – Attachment and attention-seeking

Most dogs are strongly attached to their people, and some demand a great deal of attention and affection from them. Thinking back over the recent past, how often has your dog shown each of the following signs of attachment or attention-seeking.

Where 'Never' = 0, 'Seldom' = 1, 'Sometimes' = 2, 'Usually' = 3 and 'Always' = 4

68. Displays a strong attachment for one particular member of the household.
69. Tends to follow you (or other members of the household) about the house, from room to room.
70. Tends to sit close, or in contact with, you (or others), when you are sitting down.
71. Tends to nudge, nuzzle or paw you (or others) for attention when you are sitting down.
72. Becomes agitated (whines, jumps up, tries to intervene) when you (or others) show affection for another person.
73. Becomes agitated (whines, jumps up, tries to intervene) when you (or others) show affection for another dog or animal.

Section 7 – Miscellaneous

Dogs display a wide range of miscellaneous behavior problems in addition to those already covered by this questionnaire. Thinking back over the recent past, please indicate how often your dog has shown any of the following behaviors:

Where 'Never' = 0, 'Seldom' = 1, 'Sometimes' = 2, 'Usually' = 3 and 'Always' = 4

74. Chases or would chase cats given the opportunity.
75. Chases or would chase birds given the opportunity.
76. Chases or would chase squirrels, rabbits and other small animals given the opportunity.
77. Escapes or would escape from home or yard given the chance.
78. Rolls in animal dropping or other 'smelly' substances.
79. Eats own or other animals' droppings or feces.
80. Chews inappropriate objects.
81. 'Mounts' objects, furniture or people.
82. Begs persistently for food when people are eating.
83. Steals food.
84. Nervous or frightened on stairs.
85. Pulls excessively hard when on the leash.
86. Urinates against objects/furnishing in your home.
87. Urinates when approached, petted, handled or picked up.
88. Urinates when left alone at night, or during the daytime.
89. Defecates when left alone at night, or during the daytime.
90. Hyperactive, restless, has trouble settling down.
91. Playful, puppyish, boisterous.
92. Active, energetic, always on the go.
93. Stares intently at nothing visible.
94. Snaps at (invisible) flies.
95. Chases own tail/hind end.
96. Chases/follows shadows, light spots, etc.
97. Barks persistently when alarmed or excited.
98. Licks him/herself excessively.
99. Licks people or objects excessively.

100. Displays other bizarre, strange or repetitive behavior(s)*.
*Please describe briefly:

From the 100 items shown above, 78 are used to score the 14 categories or C-BARQ behavioral traits that were later used for GWAS analysis. The behavioral traits calculations are shown below. The remaining "Miscellaneous" questions are scored individually and do not have an overall score.

- “Stranger-directed aggression” score = (questions 10 + 11 + 12 + 15 + 16 + 18 + 20 + 21 + 22 + 28)/10.
- “Owner-directed aggression” score = (questions 9 + 13 + 14 + 17 + 19 + 25 + 30 + 31)/8.
- “Dog-directed aggression” score = (questions 23 + 24 + 26 + 29)/4.
- “Dog-directed fear” score = (questions 45 + 46 + 52 + 53)/4.
- “Dog rivalry” score = (questions 32 + 33 + 34 + 35)/4.
- “Trainability” score = (questions 1 + 2 + 3 + 4 + 5 + 6 + 7 + 8)/8.
- “Chasing” score = (questions 27 + 74 + 75 + 76)/4.
- “Stranger-directed fear” score = (questions 36 + 37 + 39 + 40)/4.
- “Nonsocial fear” score = (questions 38 + 41 + 42 + 44 + 47 + 48)/6.
- “Separation-related problems” score = (questions 54 + 55 + 56 + 57 + 58 + 59 + 60 + 61)/8.
- “Touch sensitivity” score = (questions 43 + 49 + 50 + 51)/4.
- “Excitability” score = (questions 62 + 63 + 64 + 65 + 66 + 67)/6.
- “Attachment/attention-seeking” score = (questions 68 + 69 + 70 + 71 + 72 + 73)/6.
- “Energy” score = (questions 91 + 92)/2.

If respondents were unable to answer a question, they had the option to select 'Not observed/Not applicable', which appeared as blank values in the dataset. When this option was selected, the factors were scored by calculating the average of the remaining scores. When more than 25% of the questions loading into a factor were blank (answered as 'Not observed/Not applicable'), the overall factor score was recorded as a blank value.

Table S2. Thresholds used to define cases and controls for relevant analyses, and comparative summary statistics for case and control groups. Count, number of dogs in case or control group; M, Male; F, female; Disease, C-BARQ question (any disease that might or might not result in specific medical treatment: yes, no); Service dog, vs pet/companion dog; Activity, C-BARQ question, low, medium, high coded 1-3 respectively; SD – standard deviation.

C-BARQ Trait	Group	n Count	Sex		Neutered		Disease		Service dog		Age (years)				Activity			
			F	M	Yes	No	Yes	No	Yes	No	Mean	SD	Min	Max	Mean	SD	Min	Max
Dog-directed aggression	Control	455	261	194	385	70	335	120	5	450	4.81	1.09	3	7	2.15	0.49	1	3
	Cases	145	51	94	118	27	109	36	1	144	4.95	1.13	3	7	2.30	0.57	1	3
Dog-directed fear	Control	355	67	61	117	11	96	32	0	128	4.79	1.09	3	7	2.24	0.56	1	3
	Cases	128	162	193	276	79	256	99	4	351	4.87	1.11	3	7	2.24	0.50	1	3
Dog rivalry	Control	824	102	104	171	35	156	50	0	206	4.81	1.13	3	7	2.25	0.53	1	3
	Cases	206	406	418	687	137	600	224	12	812	4.81	1.09	3	7	2.19	0.52	1	3
Energy	Control	939	464	475	789	150	697	242	10	929	4.84	1.10	3	7	2.15	0.50	1	3
	Cases	248	120	128	197	51	179	69	3	245	4.69	1.05	3	7	2.42	0.54	1	3
Non-social fear	Control	281	146	135	215	66	197	84	5	276	4.88	1.06	3	7	2.26	0.52	1	3
	Cases	155	80	75	141	14	129	26	3	152	4.72	1.15	3	7	2.12	0.47	1	3
Owner-directed aggression	Control	488	245	243	395	93	344	144	5	483	4.82	1.13	3	7	2.23	0.55	1	3
	Cases	122	59	63	103	19	95	27	2	120	4.81	1.06	3	7	2.25	0.53	1	3
Separation related problems	Control	512	248	264	424	88	372	140	3	509	4.86	1.13	3	7	2.17	0.51	1	3
	Cases	128	47	81	110	18	93	35	3	125	4.77	1.10	3	7	2.16	0.50	1	3
Stranger-directed aggression	Control	507	75	66	119	22	110	31	3	138	4.77	1.14	3	7	2.26	0.56	1	3
	Cases	141	257	250	415	92	381	126	7	500	4.85	1.11	3	7	2.17	0.51	1	3
Stranger-directed fear	Control	660	314	346	540	120	485	175	9	651	4.85	1.10	3	7	2.22	0.54	1	3
	Cases	165	100	65	146	19	130	35	2	163	4.61	1.12	3	7	2.17	0.50	1	3
Touch sensitivity	Control	532	95	76	156	15	136	35	1	170	4.94	1.11	3	7	2.12	0.53	1	3
	Cases	171	266	266	426	106	386	146	4	528	4.82	1.10	3	7	2.24	0.50	1	3
Attachment and attention seeking		1187	584	603	986	201	876	311	13	1174	4.81	1.09	3	7	2.2	0.52	1	3
Chasing		1187	584	603	986	201	876	311	13	1174	4.81	1.09	3	7	2.2	0.52	1	3
Excitability		1187	584	603	986	201	876	311	13	1174	4.81	1.09	3	7	2.2	0.52	1	3

Table S3: Heritability of C-BARQ behavioral traits in golden retrievers.

Heritability was calculated using the genome-based restricted maximum likelihood (GREML) approach implemented in GCTA, specifically employing the GREML-LDMS approach to adjust for the influence of linkage disequilibrium (LD) and minor allele frequency (MAF) on the estimated SNP heritability. Heritability is expressed as a percentage and standard error (SE) is shown.

Trait	Heritability (%)
**Stranger Directed Aggression**	69.0 ± 14.41
**Owner Directed Aggression**	8.87 ± 5.0
**Dog Directed Aggression**	50.51 ± 14.88
**Stranger Directed Fear**	19.38 ± 10.1
**Dog Directed Fear**	7.36 ± 12.38
**Non-social Fear**	65.61 ± 13.15
**Separation Related Problem**	32.21 ± 14.26
**Touch Sensitivity**	25.49 ± 11.64
**Dog Rivalry**	13.12 ± 5.21
**Trainability**	4.64 ± 3.12
**Chasing**	16.18 ± 5.06
**Energy Level**	18.69 ± 7.08
**Excitability**	21.61 ± 5.76
**Attachment Attention Seeking**	12.13 ± 5.29

Table S4. Candidate genes identified in the C-BARQ GWAS annotated with information regarding function, human disease associations and the phenotype of gene knock-out in the mouse. The name of each gene is followed by its abbreviated form. Proximal candidate genes (those closest to the lead SNP at each locus) are highlighted in bold, and the row is shaded. Genes that are not represented in bold and have rows that are not shaded are positional candidate genes (within our region of interest $r^2 > 0.7$). Gene function is copied from the NCBI Gene Summary. Human disorders robustly associated with the gene are extracted from the Gene Cards website (www.genecards.org) which reports MalaCards-supplied gene-disease associations (23). The impact of gene knock-out in mouse was obtained from the International Mouse Phenotype Consortium (IMPC) Phenotypes website (www.mousephenotype.org) (24) with a focus on Behavioral/Neurological and Nervous System as these were deemed to be most relevant. IMPC Phenotype for KO genes relevant to Vision/Eye are provided as significant or not as this can impact canine behavior.

Trait	Candidate Gene Name	NCBI Gene Summary	Disorders	IMPC Phenotypes (Behaviour/ Neurological)	IMPC Phenotypes (Nervous System)	IMPC Phenotypes (Vison/ Eye)
Dog-directed aggression	Protein Tyrosine Phosphate Non-Receptor Type 1 (PTPN1)	The protein encoded by this gene is the founding member of the protein tyrosine phosphatase (PTP) family, which was isolated and identified based on its enzymatic activity and amino acid sequence. PTPs catalyze the hydrolysis of the phosphate monoesters specifically on tyrosine residues. Members of the PTP family share a highly conserved catalytic motif, which is essential for the catalytic activity. PTPs are known to be signalling molecules that regulate a variety of cellular processes including cell growth, differentiation, mitotic cycle, and oncogenic transformation. This PTP has been shown to act as a negative regulator of insulin signalling by dephosphorylating the phosphotryosine residues of insulin receptor kinase. This PTP was also reported to dephosphorylate epidermal growth factor receptor kinase, as well as JAK2 and TYK2 kinases, which implicated the role of this PTP in cell growth control, and cell response to interferon stimulation. Two transcript variants encoding different isoforms have been found for this gene. [provided by RefSeq, Jul 2013]	Type 2 Diabetes Mellitus	Not tested	Not tested	Not tested
Dog-directed fear	Zinc Finger CCCH-Type Containing 12C (ZC3H12C)	Predicted to enable endoribonuclease activity and mRNA binding activity. Predicted to be involved in RNA phosphodiester bond hydrolysis, endonucleolytic. Predicted to be active in cytoplasmic ribonucleoprotein granule and nucleus. [provided by Alliance of Genome Resources, Apr 2022]	N/A	No significant impact	No significant impact	No significant impact
	Preoxiredoxin 1 (PRDX1)	This gene encodes a member of the peroxiredoxin family of antioxidant enzymes, which reduce hydrogen peroxide and alkyl hydroperoxides. The encoded protein may play an antioxidant protective role in cells, and may contribute to the antiviral activity of CD8(+) T-cells. This protein may have a proliferative effect and play a role in cancer development or progression. Four transcript variants encoding the same protein have been identified for this gene. [provided by RefSeq, Jan 2011]	Methylmalonic aciduria and homocystinuria, methylmalonic acidaemia, abdominal obesity-metabolic syndrome, Opitz-Kaveggia Syndrome, epilepsy.	Not tested	Not tested	Not tested
	Hormonally Upregulated Neu-Associated Kinase (HUNK)	Predicted to enable protein serine/threonine kinase activity. Predicted to be involved in intracellular signal transduction and protein phosphorylation. [provided by Alliance of Genome Resources, Apr 2022]	Recombinase activating gene 1 deficiency, immunodeficiency 39, combined cellular and humoral immune defects with granulomas, Bardet-Biedl Syndrome, Baylisascariasis.	No significant impact	Decreased prepulse inhibition	Not tested

Trait	Candidate Gene Name	NCBI Gene Summary	Disorders	IMPC Phenotypes (Behaviour/ Neurological)	IMPC Phenotypes (Nervous System)	IMPC Phenotypes (Vison/ Eye)
	Leucine-Rich Repeat Containing 41 (LRRC41)	Predicted to enable identical protein binding activity. Predicted to be involved in protein ubiquitination. Located in membrane. [provided by Alliance of Genome Resources, Apr 2022]	Primary ovarian insufficiency	Abnormal vocalization, decreased vertical activity, hyperactivity, increased startle reflex.	No significant impact	No significant impact
	RAD54 Like (RAD54L)	The protein encoded by this gene belongs to the DEAD-like helicase superfamily, and shares similarity with Saccharomyces cerevisiae Rad54, a protein known to be involved in the homologous recombination and repair of DNA. This protein has been shown to play a role in homologous recombination related repair of DNA double-strand breaks. The binding of this protein to double-strand DNA induces a DNA topological change, which is thought to facilitate homologous DNA paring, and stimulate DNA recombination. Alternative splicing results in multiple transcript variants encoding the same protein.[provided by RefSeq, Dec 2008]	Non-Hodgkin Familial Lymphoma, Breast Ductal Carcinoma, Colon Adenocarcinoma, primary ovarian insufficiency, breast cancer	No significant impact	No significant impact	Significant impact
	Leucine-Rich Adaptor protein 1 (LURAP1)	Involved in positive regulation of I-kappaB kinase/NF-kappaB signaling and positive regulation of cytokine production. Located in cytosol and intracellular membrane-bounded organelle. [provided by Alliance of Genome Resources, Apr 2022]	N/A	Increased grip strength	No significant impact	No significant impact
	Protein O-Like Mannose Beta 1,2-N-acetylglucoseaminyltransferase (POMGNT1)	This gene encodes a type II transmembrane protein that resides in the Golgi apparatus. It participates in O-mannosyl glycosylation and is specific for alpha linked terminal mannose. Mutations in this gene may be associated with muscle-eye-brain disease and several congenital muscular dystrophies. Alternatively spliced transcript variants that encode different protein isoforms have been described. [provided by RefSeq, Feb 2014]	Muscular Dystrophy-Dystroglycanopathy, Rhinitis Pigmentosa, Muscle-Eye-Brain Disease	Not tested	Not tested	Not tested
	Tetraspanin 1 (TSPAN1)	The protein encoded by this gene is a member of the transmembrane 4 superfamily, also known as the tetraspanin family. Most of these members are cell-surface proteins that are characterized by the presence of four hydrophobic domains. The proteins mediate signal transduction events that play a role in the regulation of cell development, activation, growth and motility. [provided by RefSeq, Jul 2008]	Muscle-Eye-Brain Disease, muscular distrophy-dystronglycanopathy.	Not tested	Not tested	Not tested
	Microtubule Associated Serine/Threonine kinase 2 (MAST2)	Enables phosphatase binding activity. Predicted to be involved in several processes, including peptidyl-serine phosphorylation; regulation of interleukin-12 production; and spermatid differentiation. Predicted to be located in cytoplasm and plasma membrane. Predicted to be active in microtubule cytoskeleton. [provided by Alliance of Genome Resources, Apr 2022]	Haematologic cancer	No significant impact	No significant impact	No significant impact
	Intracisternal A Particle-Promoted Polypeptide (IPP)	The protein encoded by this gene is a member of the kelch family of proteins, which is characterized by a 50 amino acid repeat which interacts with actin. Transcript variants have been described but their full-length nature has not been determined. [provided by RefSeq, Jul 2008]	N/A	N/A	N/A	N/A
	GC-Rich Promoter Binding Protein 1-Like 1 (GPBP1L1)	Predicted to enable DNA binding activity and RNA binding activity. Predicted to be involved in regulation of transcription, DNA-templated. Predicted to be active in nucleus. [provided by Alliance of Genome Resources, Apr 2022]	N/A	No significant impact	No significant impact	No significant impact

Trait	Candidate Gene Name	NCBI Gene Summary	Disorders	IMPC Phenotypes (Behaviour/ Neurological)	IMPC Phenotypes (Nervous System)	IMPC Phenotypes (Vison/ Eye)
	Coiled-Coil Domain Containing 17 (CCDC17)	N/A	N/A	Not tested	Not tested	Not tested
	Nuclear Autoantigenic Sperm Protein (NASP)	This gene encodes a H1 histone binding protein that is involved in transporting histones into the nucleus of dividing cells. Multiple isoforms are encoded by transcript variants of this gene. The somatic form is expressed in all mitotic cells, is localized to the nucleus, and is coupled to the cell cycle. The testicular form is expressed in embryonic tissues, tumor cells, and the testis. In male germ cells, this protein is localized to the cytoplasm of primary spermatocytes, the nucleus of spermatids, and the periacrosomal region of mature spermatozoa. [provided by RefSeq, Jul 2008]	Spastic paraplegia 16 (X-Linked), factitious disorder, stuttering.	Not tested	Not tested	Not tested
	Metabolism of Cobalamin Associated C (MMACHC)	The exact function of the protein encoded by this gene is not known, however, its C-terminal region shows similarity to TonB, a bacterial protein involved in energy transduction for cobalamin (vitamin B12) uptake. Hence, it is postulated that this protein may have a role in the binding and intracellular trafficking of cobalamin. Mutations in this gene are associated with methylmalonic aciduria and homocystinuria type cblC. [provided by RefSeq, Oct 2009]	Methylmalonic acidaemia, aciduria and homocystinuria, disorders of intracellular cobalamin metabolism.	N/A	N/A	N/A
	Target of EGR1 (TOE1)	Enables poly(A)-specific ribonuclease activity and snRNA binding activity. Involved in RNA phosphodiester bond hydrolysis, exonucleolytic and snRNA 3'-end processing. Located in Cajal body and cytoplasm. Implicated in pontocerebellar hypoplasia type 7. [provided by Alliance of Genome Resources, Apr 2022]	Pontocerebellar hypoplasia, familial adenomatous polyposis, inherited cancer-predisposing syndrome, gastric cancer.	Limp grasping	No significant impact	No significant impact
	mutY DNA Glycosylase (MUTYH)	This gene encodes a DNA glycosylase involved in oxidative DNA damage repair. The enzyme excises adenine bases from the DNA backbone at sites where adenine is inappropriately paired with guanine, cytosine, or 8-oxo-7,8-dihydroguanine, a major oxidatively damaged DNA lesion. The protein is localized to the nucleus and mitochondria. This gene product is thought to play a role in signaling apoptosis by the introduction of single-strand breaks following oxidative damage. Mutations in this gene result in heritable predisposition to colorectal cancer, termed MUTYH-associated polyposis (MAP). Multiple transcript variants encoding different isoforms have been found for this gene. [provided by RefSeq, Apr 2017]	Familial adenomatous polyposis, gastric cancer, pilomatrixoma, familial colorectal cancer type X.	Not tested	Not tested	Not tested
	4-Hydroxyphenylpyruvate Dioxygenase-Like (HPDL)	The protein encoded by this intronless gene localizes to mitochondria, where it may function as 4-hydroxyphenylpyruvate dioxygenase. Clinical studies have identified several bi-allelic variants in this gene that lower the level of the encoded protein and lead to a clinically variable form of pediatric-onset spastic movement disorder. [provided by RefSeq, Aug 2020]	Neurodevelopmental disorder with progressive spasticity and brain white matter abnormalities, spastic paraplegia 83, spastic ataxia, non-specific Syndromic Intellectual Disability.	Abnormal behaviour, decreased anxiety-related response, decreased thigmotaxis, no spontaneous movement.	Decreased prepulse inhibition	Significant impact

Trait	Candidate Gene Name	NCBI Gene Summary	Disorders	IMPC Phenotypes (Behaviour/ Neurological)	IMPC Phenotypes (Nervous System)	IMPC Phenotypes (Vison/ Eye)
	Uroporphyrignogen Decarboxylase (UROD)	This gene encodes an enzyme in the heme biosynthetic pathway. This enzyme is responsible for catalyzing the conversion of uroporphyrinogen to coproporphyrinogen through the removal of four carboxymethyl side chains. Mutations and deficiency in this enzyme are known to cause familial porphyria cutanea tarda and hepatoerythropoetic porphyria.[provided by RefSeq, Aug 2010]	Porphyria.	Not tested	Not tested	Not tested
	HECT Domain E3 Ubiquitin Protein Ligase 3 (HECTD3)	The protein encoded by this gene transfers ubiquitin from an E2 ubiquitin-conjugating enzyme to targeted substrates, leading to the degradation of those substrates. The encoded protein has been shown to transfer ubiquitin to TRIOBP to facilitate cell cycle progression, and to STX8. [provided by RefSeq, Dec 2012]	N/A	Decreased grip strength	Decreased brain size	Not tested
	Eukaryotic Translation Initiation Factor 2B, Subunit 3 Gamma (EIF2B3)	The protein encoded by this gene is one of the subunits of initiation factor eIF2B, which catalyzes the exchange of eukaryotic initiation factor 2-bound GDP for GTP. It has also been found to function as a cofactor of hepatitis C virus internal ribosome entry site-mediated translation. Mutations in this gene have been associated with leukodystrophy with vanishing white matter. Alternatively spliced transcript variants encoding different isoforms have been found for this gene. [provided by RefSeq, Oct 2009]	Leukoencephalopathy with vanishing white matter, Pelizaeus-Merzbacher Disorder.	No significant impact	No significant impact	No significant impact
	Zinc Finger SWIM-Type Containing 5 (ZSWIM5)	Predicted to enable zinc ion binding activity. Located in extracellular space. [provided by Alliance of Genome Resources, Apr 2022]	N/A	No significant impact	No significant impact	No significant impact
	Transmembrane Protein 69 (TMEM69)	Predicted to be integral component of membrane. [provided by Alliance of Genome Resources, Apr 2022]	N/A	Not tested	Not tested	Not tested
	Pik3r3 Upstream Reading Frame (P3R3URF)	Predicted to enable 1-phosphatidylinositol-3-kinase regulator activity. Predicted to be involved in phosphatidylinositol phosphate biosynthetic process. Predicted to be part of phosphatidylinositol 3-kinase complex. [provided by Alliance of Genome Resources, Apr 2022]	N/A	Not tested	Not tested	Not tested
**Non-social fear**	**Fibulin 1 (FBLN1)**	Fibulin 1 is a secreted glycoprotein that becomes incorporated into a fibrillar extracellular matrix. Calcium-binding is apparently required to mediate its binding to laminin and nidogen. It mediates platelet adhesion via binding fibrinogen. Four splice variants which differ in the 3' end have been identified. Each variant encodes a different isoform, but no functional distinctions have been identified among the four variants. [provided by RefSeq, Jul 2008]	Synpolydactyly, syndactyly, brachydactyly, bone disease	Not tested	Not tested	Not tested
	**Activating Signal Cointegrator 1 Complex Subunit 3 (ASCC3)**	This gene encodes a protein that belongs to a family of helicases that are involved in the ATP-dependent unwinding of nucleic acid duplexes. The encoded protein is the largest subunit of the activating signal cointegrator 1 complex that is involved in DNA repair and resistance to alkylation damage. Alternate splicing results in multiple transcript variants. [provided by RefSeq, Sep 2013]	Intellectual developmental disorder autism spectrum disorder, congenital myopathy,	Increased startle reflex	No significant impact	Significant impact
	**Kelch Repeat and BTB (POZ) Domain Containing 8 (KBTBD8)**	Involved in neural crest cell development; neural crest formation; and protein monoubiquitination. Part of Cul3-RING ubiquitin ligase complex. [provided by Alliance of Genome Resources, Apr 2022]	N/A	No significant impact	No significant impact	No significant impact

Trait	Candidate Gene Name	NCBI Gene Summary	Disorders	IMPC Phenotypes (Behaviour/ Neurological)	IMPC Phenotypes (Nervous System)	IMPC Phenotypes (Vison/ Eye)
	Ataxin 10 (ATXN10)	This gene encodes a protein that may function in neuron survival, neuron differentiation, and neuritogenesis. These roles may be carried out via activation of the mitogen-activated protein kinase cascade. Expansion of an ATTCT repeat from 9-32 copies to 800-4500 copies in an intronic region of this locus has been associated with spinocerebellar ataxia, type 10. Alternatively spliced transcript variants have been [provided by RefSeq, Jul 2016]	Spinocerebellar ataxia, Machado-Joseph Disease, X-linked sideroblastic anaemia with ataxia, Limb-Girdle muscular dystrophy.	No significant impact	No significant impact	No significant impact
**Stranger-directed fear**	**Adenylate Cyclase Activating polypeptide 1 (ADCYAP1)**	This gene encodes a small nuclear ribonucleoprotein that belongs to the SNRNP core protein family. The protein may act as a charged protein scaffold to promote SNRNP assembly or strengthen SNRNP-SNRNP interactions through nonspecific electrostatic contacts with RNA. Two transcript variants encoding different isoforms have been found for this gene. [provided by RefSeq, May 2014]	Systemic lupus erythematosus, autoimmune diseases, Noonan Syndrome 2, muscular atrophy.	Not tested	Not tested	Not tested
	**Adducin 2 (ADD2)**	Adducins are heteromeric proteins composed of different subunits referred to as adducin alpha, beta and gamma. The three subunits are encoded by distinct genes and belong to a family of membrane skeletal proteins involved in the assembly of spectrin-actin network in erythrocytes and at sites of cell-cell contact in epithelial tissues. While adducins alpha and gamma are ubiquitously expressed, the expression of adducin beta is restricted to brain and hematopoietic tissues. Adducin, originally purified from human erythrocytes, was found to be a heterodimer of adducins alpha and beta. Polymorphisms resulting in amino acid substitutions in these two subunits have been associated with the regulation of blood pressure in an animal model of hypertension. Heterodimers consisting of alpha and gamma subunits have also been described. Structurally, each subunit is comprised of two distinct domains. The amino-terminal region is protease resistant and globular in shape, while the carboxy-terminal region is protease sensitive. The latter contains multiple phosphorylation sites for protein kinase C, the binding site for calmodulin, and is required for association with spectrin and actin. Alternatively spliced transcript variants have been described. [provided by RefSeq, Jun 2010]	Hereditary spherocytosis and elliptocytosis, hypertension and Lautenbacher's Syndrome.	Not tested	Not tested	Not tested
	**Solute Carrier Family 35, Member B3 (SLC35B3)**	This gene is a member of the solute carrier family. The encoded protein is involved in the transport of 3-prime phosphoadenosine 5-prime phosphosulfate (PAPS) from the nucleus or the cytosol to the Golgi lumen. This gene has been reported to be expressed preferentially in the human colon tissues. Alternative splicing results in multiple transcript variants. [provided by RefSeq, Dec 2013]	Chondrodysplasia with joint dislocations, Thiamine-responsive megaloblastic anaemia Syndrome, chromosome 6Pter-P24 Deletion Syndrome, keratosis palmoplantaris striata, hereditary multiple exostoses	Not tested	Not tested	Not tested
	Small Nuclear Ribonucleoprotein D1 (SNRPD1)	This gene encodes a small nuclear ribonucleoprotein that belongs to the SNRNP core protein family. The protein may act as a charged protein scaffold to promote SNRNP assembly or strengthen SNRNP-SNRNP interactions through nonspecific electrostatic contacts with RNA. Two transcript variants encoding different isoforms have been found for this gene. [provided by RefSeq, May 2014]	Systemic lupus erythematosus, autoimmune diseases, Noonan Syndrome 2, muscular atrophy.	Not tested	Not tested	Not tested

Trait	Candidate Gene Name	NCBI Gene Summary	Disorders	IMPC Phenotypes (Behaviour/ Neurological)	IMPC Phenotypes (Nervous System)	IMPC Phenotypes (Vison/ Eye)
	Establishment of Sister Chromatid Cohesion N-acetyltransferase 1 (ESCO1)	Enables identical protein binding activity; peptide-lysine-N-acetyltransferase activity; and zinc ion binding activity. Involved in peptidyl-lysine acetylation; post-translational protein acetylation; and regulation of DNA replication. Located in chromatin. [provided by Alliance of Genome Resources, Apr 2022]	1,4-phenylenediamine allergic contact dermatitis, Roberts-Sc Phocomelia Syndrome, mucopolysaccharidosis, trimethoprim allergy, ceftazidime allergy.	No significant impact	No significant impact	No significant impact
	GERB1 Like Retinoic Acid Receptor Coactivator (GREB1L)	Acts upstream of or within kidney development. Predicted to be integral component of membrane. Implicated in autosomal dominant nonsyndromic deafness and renal agenesis. [provided by Alliance of Genome Resources, Apr 2022]	Renal hypodysplasia/aplasia, deafness, Mayer-Rokitansky-Kuster-Hauser Syndrome, Mullerian duct aplasia, unilateral renal agenesis, cervicothoracic Somite anomalies.	N/A	N/A	N/A
	Rho-Associated Coiled-Coil Containing protein Kinase 1 (ROCK1)	This gene encodes a protein serine/threonine kinase that is activated when bound to the GTP-bound form of Rho. The small GTPase Rho regulates formation of focal adhesions and stress fibers of fibroblasts, as well as adhesion and aggregation of platelets and lymphocytes by shuttling between the inactive GDP-bound form and the active GTP-bound form. Rho is also essential in cytokinesis and plays a role in transcriptional activation by serum response factor. This protein, a downstream effector of Rho, phosphorylates and activates LIM kinase, which in turn, phosphorylates cofilin, inhibiting its actin-depolymerizing activity. A pseudogene, related to this gene, is also located on chromosome 18. [provided by RefSeq, Aug 2015]	Brest and lung cancer	No significant impact	No significant impact	No significant impact
	Ubiquitin Specific peptidase 14 (USP14)	This gene encodes a member of the ubiquitin-specific processing (UBP) family of proteases that is a deubiquitinating enzyme (DUB) with His and Cys domains. This protein is located in the cytoplasm and cleaves the ubiquitin moiety from ubiquitin-fused precursors and ubiquitinylated proteins. Mice with a mutation that results in reduced expression of the ortholog of this protein are retarded for growth, develop severe tremors by 2 to 3 weeks of age followed by hindlimb paralysis and death by 6 to 10 weeks of age. Alternate transcriptional splice variants, encoding different isoforms, have been characterized. [provided by RefSeq, Jul 2008]	Manchado-Joseph Disease, cerebellar ataxia, multiple myeloma, frontotemporal dementia and/or myotrophin lateral sclerosis 7, spinocerebellar ataxia.	No significant impact	No significant impact	No significant impact
	THO Complex Subunit 1 (THOC1)	Predicted to enable DNA binding activity and RNA binding activity. Involved in several processes, including negative regulation of DNA damage checkpoint; regulation of nucleobase-containing compound metabolic process; and viral mRNA export from host cell nucleus. Located in cytoplasm and nuclear speck. Part of THO complex part of transcription export complex. Colocalizes with chromosome, telomeric region. [provided by Alliance of Genome Resources, Apr 2022]	Sarcoma, retinoblastoma, sensorineural hearing loss	No significant impact	No significant impact	No significant impact
	Collection Subfamily Member 12 (COLEC12)	This gene encodes a member of the C-lectin family, proteins that possess collagen-like sequences and carbohydrate recognition domains. This protein is a scavenger receptor that displays several functions associated with host defense. It can bind to carbohydrate antigens on microorganisms, facilitating their recognition and removal. It also mediates the recognition, internalization, and degradation of oxidatively modified low density lipoprotein by vascular endothelial cells. [provided by RefSeq, May 2018]	Intellectual developmental disorder, Ehlers-Danlos Syndrome (hypermotility type), autism spectrum disorder.	Increased startle reflex, hyperactivity.	Decreased prepulse inhibition, hydrocephaly.	No significant impact

Trait	Candidate Gene Name	NCBI Gene Summary	Disorders	IMPC Phenotypes (Behaviour/ Neurological)	IMPC Phenotypes (Nervous System)	IMPC Phenotypes (Vison/ Eye)
	Centrin 1 (CETN1)	The protein encoded by this gene plays important roles in the determination of centrosome position and segregation, and in the process of microtubule severing. This protein is localized to the centrosome of interphase cells, and redistributes to the region of the spindle poles during mitosis, reflecting the dynamic behavior of the centrosome during the cell cycle. [provided by RefSeq, Jan 2015]	Intellectual developmental disorder 40, congenital stationary night blindness, male infertility, primary ciliary dyskinesia.	Not tested	Not tested	Not tested
	Clusterin like 1 (CLUL1)	Predicted to enable misfolded protein binding activity. Predicted to be located in extracellular region. Predicted to be active in extracellular space and nucleus. [provided by Alliance of Genome Resources, Apr 2022]	Chromosome 18P Deletion Syndrome, myopia, epidermolysis bullosa simplex 2F, autism spectrum disorder	N/A	N/A	N/A
	Thymidylate Synthase (TYMS)	Thymidylate synthase catalyzes the methylation of deoxyuridylate to deoxythymidylate using, 10-methylenetetrahydrofolate (methylene-THF) as a cofactor. This function maintains the dTMP (thymidine-5-prime monophosphate) pool critical for DNA replication and repair. The enzyme has been of interest as a target for cancer chemotherapeutic agents. It is considered to be the primary site of action for 5-fluorouracil, 5-fluoro-2-prime-deoxyuridine, and some folate analogs. Expression of this gene and that of a naturally occurring antisense transcript, mitochondrial enolase superfamily member 1 (GeneID:55556), vary inversely when cell-growth progresses from late-log to plateau phase. Polymorphisms in this gene may be associated with etiology of neoplasia, including breast cancer, and response to chemotherapy. [provided by RefSeq, Aug 2017]	Dyskeratosis congenita	No significant impact	No significant impact	Not tested
	Enolase Superfamily Member (ENOSF1)	This gene can encode a mitochondrial enzyme that is thought to convert L-fuconate to 2-keto-3-deoxy-L-fuconate. This locus was originally identified as the source of antisense RNAs of the adjacent thymidylate synthase gene. Splice variants at this locus may contain an alternate 3' exon that is complementary to the 3'UTR and terminal intron of the thymidylate synthase (TS) RNA and may downregulate TS expression. [provided by RefSeq, Aug 2017]	Chromosome 18P Deletion Syndrome, aplastic anaemia, autism spectrum disorder	N/A	N/A	N/A
	YES Proto-Oncogene 1 (YES1)	This gene is the cellular homolog of the Yamaguchi sarcoma virus oncogene. The encoded protein has tyrosine kinase activity and belongs to the src family of proteins. This gene lies in close proximity to thymidylate synthase gene on chromosome 18, and a corresponding pseudogene has been found on chromosome 22. [provided by RefSeq, Jul 2008]	Sarcoma, colorectal cancer, Lynch Syndrome 2, rhabdomyosarcoma, breast cancer	Not tested	Not tested	Not tested
	Folliculogenesis Specific Basic Helix-Loop-Helix (FIGLA)	This gene encodes a protein that functions in postnatal oocyte-specific gene expression. The protein is a basic helix-loop-helix transcription factor that regulates multiple oocyte-specific genes, including genes involved in folliculogenesis and those that encode the zona pellucida. Mutations in this gene cause premature ovarian failure type 6. [provided by RefSeq, Sep 2009]	Premature ovarian failure, primary ovarian insufficiency.	Not tested	Not tested	Not tested
	C-Type Lectin Domain Family 4, Member F (CLEC4F)	Predicted to enable galactose binding activity and glycolipid binding activity. Predicted to be involved in endocytosis. Predicted to act upstream of or within NK T cell activation. Predicted to be located in plasma membrane. [provided by Alliance of Genome Resources, Apr 2022]	Cystic echinococcosis, lysosomal storage disease	Not tested	Not tested	Not tested

Trait	Candidate Gene Name	NCBI Gene Summary	Disorders	IMPC Phenotypes (Behaviour/ Neurological)	IMPC Phenotypes (Nervous System)	IMPC Phenotypes (Vison/ Eye)
	Transforming Growth Factor Alpha (TGFA)	This gene encodes a growth factor that is a ligand for the epidermal growth factor receptor, which activates a signaling pathway for cell proliferation, differentiation and development. This protein may act as either a transmembrane-bound ligand or a soluble ligand. This gene has been associated with many types of cancers, and it may also be involved in some cases of cleft lip/palate. Alternatively spliced transcript variants encoding different isoforms have been found for this gene. [provided by RefSeq, Sep 2011]	Tooth agenesis	Not tested	Not tested	Not tested
	Small Nuclear Ribonucleoprotein Polypeptide G (SNRPG)	The protein encoded by this gene is a component of the U1, U2, U4, and U5 small nuclear ribonucleoprotein complexes, precursors of the spliceosome. The encoded protein may also be a part of the U7 small nuclear ribonucleoprotein complex, which participates in the processing of the 3' end of histone transcripts. Several transcript variants encoding different isoforms have been found for this gene. [provided by RefSeq, Nov 2015]	N/A	Not tested	Not tested	Not tested
	Prenylcysteine Oxidase 1 (PCYOX1)	Prenylcysteine is released during the degradation of prenylated proteins. PCYOX1 catalyzes the degradation of prenylcysteine to yield free cysteines and a hydrophobic isoprenoid product (Tschantz et al., 1999 [PubMed 10585463]).[supplied by OMIM, Mar 2008]	N/A	Not tested	Not tested	Not tested
	Cytotoxic Granule-Associated RNA Binding Protein 1 (TIA1)	The product encoded by this gene is a member of a RNA-binding protein family and possesses nucleolytic activity against cytotoxic lymphocyte (CTL) target cells. It has been suggested that this protein may be involved in the induction of apoptosis as it preferentially recognizes poly(A) homopolymers and induces DNA fragmentation in CTL targets. The major granule-associated species is a 15-kDa protein that is thought to be derived from the carboxyl terminus of the 40-kDa product by proteolytic processing. Alternative splicing resulting in different isoforms has been found for this gene. [provided by RefSeq, May 2017]	Welander Distal Myopathy, amyotrophic lateral sclerosis and/ort frontotemporal dementia, motor neuron disease, distal myopathy.	No significant impact	No significant impact	No significant impact
	Chromosome 10 C2orf42 Homolog (C10H2orf42)	N/A	N/A	N/A	N/A	N/A
Energy	**Pro-Melanin-Concentrating Hormone (PMCH)**	This gene encodes a preproprotein that is proteolytically processed to generate multiple protein products. These products include melanin-concentrating hormone (MCH), neuropeptide-glutamic acid-isoleucine (NEI), and neuropeptide-glycine-glutamic acid (NGE). Melanin-concentrating hormone is a 19-amino acid neuropeptide that stimulates hunger and may additionally regulate energy homeostasis, reproductive function, and sleep. Pseudogenes of this gene have been identified on chromosome 5. [provided by RefSeq, Jul 2015]	Familial temporal lobe epilepsy, spermatogenic failure 19, Von Economos Disease, leptin deficiency or dysfunction, Huntington Disease	No significant impact	No significant impact	No significant impact
	Anoctamin 4 (ANO4)	Enables intracellular calcium activated chloride channel activity. Involved in chloride transport. Located in plasma membrane. [provided by Alliance of Genome Resources, Apr 2022]	Generalized epilepsy with febrile seizure plus, developmental and epileptic encephalopathy, early infantile epileptic encephalopathy, temporal lobe epilepsy	Hyperactivity	No significant impact	No significant impact
	Solute Carrier Family 5 (iodine Transporter), Member 8 (SLC5A8)	SLC5A8 has been shown to transport iodide by a passive mechanism (Rodriguez et al., 2002 [PubMed 12107270]) and monocarboxylates and short-chain fatty acids by a sodium-coupled mechanism (Gopal et al., 2004 [PubMed 15322102]). In kidney, SLC5A8 functions as a high-affinity sodium-coupled lactate transporter involved in reabsorption of lactate and	N/A	Not tested	Not tested	Not tested

Trait	Candidate Gene Name	NCBI Gene Summary	Disorders	IMPC Phenotypes (Behaviour/ Neurological)	IMPC Phenotypes (Nervous System)	IMPC Phenotypes (Vison/ Eye)
		maintenance of blood lactate levels (Thangaraju et al., 2006 [PubMed 16873376]).[supplied by OMIM, Dec 2008]				
	UTP20 Small Subunit processome Component (UTP20)	UTP20 is a component of the U3 small nucleolar RNA (snoRNA) (SNORD3A; MIM 180710) protein complex (U3 snoRNP) and is involved in 18S rRNA processing (Wang et al., 2007 [PubMed 17498821]).[supplied by OMIM, Jun 2009]	N/A	Abnormal vocalization	No significant impact	No significant impact
	ADP Ribosylation Factor Like GTPasa 1 (ARL1)	The protein encoded by this gene belongs to the ARL (ADP-ribosylation factor-like) family of proteins, which are structurally related to ADP-ribosylation factors (ARFs). ARFs, described as activators of cholera toxin (CT) ADP-ribosyltransferase activity, regulate intracellular vesicular membrane trafficking, and stimulate a phospholipase D (PLD) isoform. Although, ARL proteins were initially thought not to activate CT or PLD, later work showed that they are weak stimulators of PLD and CT in a phospholipid dependent manner. Alternative splicing results in multiple transcript variants encoding different isoforms. [provided by RefSeq, Jul 2014]	N/A	Not tested	Not tested	Not tested
	Spi-C Transcription Factor (SPIC)	The protein encoded by this gene regulates the development of red pulp macrophages, which are necessary for iron homeostasis and the recycling of red blood cells. [provided by RefSeq, Aug 2016]	N/A	Not tested	Not tested	Not tested
	Myosin Binding Protein C, Slow type (MYBPC1)	This gene encodes a member of the myosin-binding protein C family. Myosin-binding protein C family members are myosin-associated proteins found in the cross-bridge-bearing zone (C region) of A bands in striated muscle. The encoded protein is the slow skeletal muscle isoform of myosin-binding protein C and plays an important role in muscle contraction by recruiting muscle-type creatine kinase to myosin filaments. Mutations in this gene are associated with distal arthrogryposis type I. Alternatively spliced transcript variants encoding multiple isoforms have been observed for this gene. [provided by RefSeq, Dec 2011]	Congenital myopathy, distal anthogenesis, Mybpc1-related autosomal recessive non-lethal Amc Syndrome, distal arthrogryposis.	Not tested	Not tested	Not tested
	N-acetylglucosamine-1-phosphate Transferase, Alpha and Beta Subunits (GNPTAB)	This gene encodes two of three subunit types of the membrane-bound enzyme N-acetylglucosamine-1-phosphotransferase, a heterohexameric complex composed of two alpha, two beta, and two gamma subunits. The encoded protein is proteolytically cleaved at the Lys928-Asp929 bond to yield mature alpha and beta polypeptides while the gamma subunits are the product of a distinct gene (GeneID 84572). In the Golgi apparatus, the heterohexameric complex catalyzes the first step in the synthesis of mannose 6-phosphate recognition markers on certain oligosaccharides of newly synthesized lysosomal enzymes. These recognition markers are essential for appropriate trafficking of lysosomal enzymes. Mutations in this gene have been associated with both mucolipidosis II and mucolipidosis IIIA.[provided by RefSeq, May 2010]	Mucolipidosis, Gnptab-related disorders, mucopolysaccharidosis	No spontaneous movement.	No significant impact	Significant impact
	DNA-damage Regulated Autophagy Modulator 1 (DRAM1)	This gene is regulated as part of the p53 tumor suppressor pathway. The gene encodes a lysosomal membrane protein that is required for the induction of autophagy by the pathway. Decreased transcriptional expression of this gene is associated with various tumors. This gene has a pseudogene on chromosome 4. [provided by RefSeq, Jul 2008]	Cone-Rod dystrophy, achromatopsia, Fuch's endothelial dystrophy, hematologic cancer.	Increased grip strength	No significant impact	No significant impact

Trait	Candidate Gene Name	NCBI Gene Summary	Disorders	IMPC Phenotypes (Behaviour/ Neurological)	IMPC Phenotypes (Nervous System)	IMPC Phenotypes (Vison/ Eye)
	Nucleoparin 37 (NUP37)	Nuclear pore complexes (NPCs) are used for transporting macromolecules between the cytoplasm and the nucleus. NPCs consist of multiple copies of 30 distinct proteins (nucleoporins), which assemble into biochemically-separable subcomplexes. The protein encoded by this gene is part of a subcomplex (Nup107-160) that is required for proper NPC function as well as for normal kinetochore-microtubule interaction and mitosis. [provided by RefSeq, Dec 2015]	Microcephaly, genetic steroid-resistant nephrotic syndrome.	Increased vertical activity	No significant impact	Significant impact
	PARP1 Binding Protein (PARPBP)	Predicted to enable DNA binding activity. Involved in negative regulation of double-strand break repair via homologous recombination. Located in chromatin and nucleoplasm. [provided by Alliance of Genome Resources, Apr 2022]	N/A	No significant impact	No significant impact	No significant impact
	Insulin-Like Growth Factor 1 (IGF1)	The protein encoded by this gene is similar to insulin in function and structure and is a member of a family of proteins involved in mediating growth and development. The encoded protein is processed from a precursor, bound by a specific receptor, and secreted. Defects in this gene are a cause of insulin-like growth factor I deficiency. Alternative splicing results in multiple transcript variants encoding different isoforms that may undergo similar processing to generate mature protein. [provided by RefSeq, Sep 2015]	Growth delay due to insulin-like growth factor 1 deficiency, insulin-like growth factor deficiency, Three M Syndrome 1, osteochondritis dissecans.	Not tested	Not tested	Not tested
	Phenylalanine Hydroxylase (PAH)	This gene encodes a member of the biopterin-dependent aromatic amino acid hydroxylase protein family. The encoded phenylalanine hydroxylase enzyme hydroxylates phenylalanine to tyrosine and is the rate-limiting step in phenylalanine catabolism. Deficiency of this enzyme activity results in the autosomal recessive disorder phenylketonuria. [provided by RefSeq, Aug 2017]	Phenylketonuria, hyperphenylalaninemia, tetrahydrobiopterin-responsive hyperphenylalaninemia/phenylketonuria, neurodevelopmental disorders with spasticity, hypomyelination leukodystrophy and brain abnormalities.	Decreased exploration in new environment	No significant impact	Significant impact
Separation related problems	**NEDD1 Gamma-Tubulin Ring Complex Targeting Factor (NEDD1)**	Predicted to be involved in protein localization to centrosome. Located in centrosome; nucleoplasm; and plasma membrane. [provided by Alliance of Genome Resources, Apr 2022]	microcephaly, congenital nervous system abnormality, lissencephaly, Seckel syndrome	N/A	N/A	N/A
	**Immunoglobulin Superfamily Member 11 (IGSF11)**	IGSF11 is an immunoglobulin (Ig) superfamily member that is preferentially expressed in brain and testis. It shares significant homology with coxsackievirus and adenovirus receptor (CXADR; MIM 602621) and endothelial cell-selective adhesion molecule (ESAM).[supplied by OMIM, Apr 2005]	N/A	Hyperactivity, impaired righting response, limb grasping.	No significant impact	No significant impact
	Vezatin Adherens Junctions Transmembrane protein (VEZT)	This gene encodes a transmembrane protein which has been localized to adherens junctions and shown to bind to myosin VIIA. Examination of expression of this gene in gastric cancer tissues have shown that expression is decreased which appears to be related to hypermethylation of the promoter. Expression of this gene may also be inhibited by binding of a specific microRNA to a target sequence in the 3' UTR of the transcripts. A pseudogene of this gene is located on the X chromosome. Alternative splicing results in multiple transcript variants. [provided by RefSeq, May 2011]	Usher Syndrome, retinitis pigmentosa	Decreased locomotor activity, decreased vertical activity	No significant impact	No significant impact

Trait	Candidate Gene Name	NCBI Gene Summary	Disorders	IMPC Phenotypes (Behaviour/ Neurological)	IMPC Phenotypes (Nervous System)	IMPC Phenotypes (Vison/ Eye)
	Methionine Aminopeptidase 2 (METAP2)	The protein encoded by this gene is a member of the methionyl aminopeptidase family. The encoded protein functions both by protecting the alpha subunit of eukaryotic initiation factor 2 from inhibitory phosphorylation and by removing the amino-terminal methionine residue from nascent proteins. Increased expression of this gene is associated with various forms of cancer, and the anti-cancer drugs fumagillin and ovalicin inhibit the protein by irreversibly binding to its active site. Inhibitors of this gene have also been shown to be effective for the treatment of obesity. A pseudogene of this gene is located on chromosome 2. Several transcript variants encoding different isoforms have been found for this gene. [provided by RefSeq, Nov 2015]	Microsporidios, liver rhabdomyosarcoma, malaria, colorectal cancer	Not tested	Not tested	Not tested
	Ubiquitin Specific Peptidase 44 (USP44)	The protein encoded by this gene is a protease that functions as a deubiquitinating enzyme. The encoded protein is thought to help regulate the spindle assembly checkpoint by preventing early anaphase onset. This protein specifically deubiquitinates CDC20, which stabilizes the anaphase promoting complex/cyclosome. [provided by RefSeq, Dec 2016]	Autism spectrum disorders	No significant impact	No significant impact	No significant impact
	Netrin 4 (NTN4)	This gene encodes a member of the netrin family of proteins, which function in various biological processes including axon guidance, tumorogenesis, and angiogenesis. Netrins are laminin-related proteins that have an N-terminal laminin-type domain, epidermal growth factor-like repeat domain, and a positively charged heparin-binding domain at the C-terminus. The protein encoded by this gene is involved in processes including neurite growth and migration, angiogenesis and mural cell adhesion to endothelial cells. Alternative splicing results in multiple transcript variants. [provided by RefSeq, Jul 2016]	Prostate cancer	No significant impact	No significant impact	Not tested
	Amidohydrolase Domain Containing 1 (AMDHD1)	Predicted to enable imidazolonepropionase activity. Predicted to be involved in histidine catabolic process. Predicted to be located in cytosol. [provided by Alliance of Genome Resources, Apr 2022]	Hemochromatosis, vitamin D-dependant Rickets	No significant impact	No significant impact	No significant impact
	Histidine Ammonia Lyase (HAL)	Histidine ammonia-lyase is a cytosolic enzyme catalyzing the first reaction in histidine catabolism, the nonoxidative deamination of L-histidine to trans-urocanic acid. Histidine ammonia-lyase defects cause histidinemia which is characterized by increased histidine and histamine and decreased urocanic acid in body fluids. Several transcript variants encoding different isoforms have been found for this gene. [provided by RefSeq, Apr 2012]	Histidinemia, intellectual developmental disorders, epilepsy	Not tested	Not tested	Not tested
	Leukotriene A4 Hydrolase (LTA4H)	The protein encoded by this gene is an enzyme that contains both hydrolase and aminopeptidase activities. The hydrolase activity is used in the final step of the biosynthesis of leukotriene B4, a proinflammatory mediator. The aminopeptidase activity has been shown to degrade proline-glycine-proline (PGP), a neutrophil chemoattractant and biomarker for chronic obstructive pulmonary disease (COPD). Several transcript variants encoding different isoforms have been found for this gene. [provided by RefSeq, Sep 2015]	Asthma, lymph node tuberculosis, psoriasis, myocardial infarction	No significant impact	No significant impact	No significant impact
	ETS Transcription Factor ELK3 (ELK3)	This gene encodes a member of the ETS-domain transcription factor family and the ternary complex factor (TCF) subfamily. Proteins in this subfamily regulate transcription when recruited by serum response factor to bind to serum response elements. This protein is activated by signal-induced phosphorylation; studies in rodents suggest that it is a transcriptional inhibitor in the absence of Ras, but activates transcription when Ras is present. Alternate splicing results in multiple transcript variants. [provided by RefSeq, Jan 2015]	N/A	No significant impact	Decreased prepulse inhibition	Significant impact

Trait	Candidate Gene Name	NCBI Gene Summary	Disorders	IMPC Phenotypes (Behaviour/ Neurological)	IMPC Phenotypes (Nervous System)	IMPC Phenotypes (Vison/ Eye)
	Cyclin Dependent Kinase 17 (CDK17)	The protein encoded by this gene belongs to the cdc2/cdkx subfamily of the ser/thr family of protein kinases. It has similarity to a rat protein that is thought to play a role in terminally differentiated neurons. Alternatively spliced transcript variants encoding different isoforms have been found. [provided by RefSeq, Jul 2010]	N/A	Hyperactivity, increased vertical activity	No significant impact	No significant impact
	Cilia and Flagella Associated Protein 54 (CFAP54)	Predicted to be involved in cilium assembly; cilium movement involved in cell motility; and spermatogenesis. Predicted to act upstream of or within cerebrospinal fluid circulation; motile cilium assembly; and mucociliary clearance. Predicted to be located in axoneme. [provided by Alliance of Genome Resources, Apr 2022]	Male infertility, coloboma of the optic nerve, primary ciliary dyskinesia, myoclonic juvenile epilepsy	Not tested	Not tested	Not tested
	Coiled-Coil Domain Containing 38 (CCDC38)	Located in centrosome. [provided by Alliance of Genome Resources, Apr 2022]	N/A	Not tested	Not tested	Not tested
Touch sensitivity	**Solute Carrier Family 35, Member F6 (SLC35F6)**	Predicted to enable transmembrane transporter activity. Involved in negative regulation of mitochondrial outer membrane permeabilization involved in apoptotic signaling pathway and positive regulation of cell population proliferation. Located in several cellular components, including lysosomal membrane; mitochondrion; and nucleoplasm. [provided by Alliance of Genome Resources, Apr 2022]	Pancreatic ductal adenocarcinoma, gingival fibromatosis, mitochondrial complex I deficiency, cerebral creatine deficiency syndrome.	No significant impact	No significant impact	No significant impact
	**Von Willebrand Factor a domain Containing 8 (VWA8)**	Predicted to enable ATP binding activity. Located in mitochondrion and peroxisome. [provided by Alliance of Genome Resources, Apr 2022]	Retinitis pigmentosa, orofacial cleft, Venezuelan haemorrhagic fever, migraine, Perrault Syndrome	No significant impact	No significant impact	Significant impact
	**Atypical Chemokine Receptor 3 (ACKR3)**	This gene encodes a member of the G-protein coupled receptor family. Although this protein was earlier thought to be a receptor for vasoactive intestinal peptide (VIP), it is now considered to be an orphan receptor, in that its endogenous ligand has not been identified. The protein is also a coreceptor for human immunodeficiency viruses (HIV). Translocations involving this gene and HMGA2 on chromosome 12 have been observed in lipomas. [provided by RefSeq, Jul 2008]	Oculomotor-abducens synkinesis, lymphoplasmacytic lymphoma, whim syndrome, ptosis, haemolytic uremic syndrome, immune deficiency diseases.	Decreased grip strength, tremors	No significant impact	Significant impact
	Family With Sequence Similarity 216, Member 16 (FAM216B)	NCBI Gene summary for FAM216B not available.	N/A	No significant impact	No significant impact	Not tested
	Tumour Necrosis Factor Superfamily, Member 11 (TNFSF11)	This gene encodes a member of the tumor necrosis factor (TNF) cytokine family which is a ligand for osteoprotegerin and functions as a key factor for osteoclast differentiation and activation. This protein was shown to be a dentritic cell survival factor and is involved in the regulation of T cell-dependent immune response. T cell activation was reported to induce expression of this gene and lead to an increase of osteoclastogenesis and bone loss. This protein was shown to activate antiapoptotic kinase AKT/PKB through a signaling complex involving SRC kinase and tumor necrosis factor receptor-associated factor (TRAF) 6, which indicated this protein may have a role in the regulation of cell apoptosis. Targeted disruption of the related gene in mice led to severe osteopetrosis and a lack of osteoclasts. The deficient mice exhibited defects in early differentiation of T and B lymphocytes, and failed to	Osteoporosis, bone disease	Not tested	Not tested	Not tested

Trait	Candidate Gene Name	NCBI Gene Summary	Disorders	IMPC Phenotypes (Behaviour/ Neurological)	IMPC Phenotypes (Nervous System)	IMPC Phenotypes (Vison/ Eye)
		form lobulo-alveolar mammary structures during pregnancy. Two alternatively spliced transcript variants have been found. [provided by RefSeq, Jul 2008]				
	A-Kinase Anchoring Protein 11 (AKAP11)	The A-kinase anchor proteins (AKAPs) are a group of structurally diverse proteins, which have the common function of binding to the regulatory subunit of protein kinase A (PKA) and confining the holoenzyme to discrete locations within the cell. This gene encodes a member of the AKAP family. The encoded protein is expressed at high levels throughout spermatogenesis and in mature sperm. It binds the RI and RII subunits of PKA in testis. It may serve a function in cell cycle control of both somatic cells and germ cells in addition to its putative role in spermatogenesis and sperm function. [provided by RefSeq, Jul 2008]	N/A	No significant impact	No significant impact	No significant impact
	Diacylglycerol Kinase (DGKH)	This gene encodes a member of the diacylglycerol kinase (DGK) enzyme family. Members of this family are involved in regulating intracellular concentrations of diacylglycerol and phosphatidic acid. Variation in this gene has been associated with bipolar disorder. Alternatively spliced transcript variants have been identified. [provided by RefSeq, Jul 2014]	Bipolar disorder, deafness, nephrolithiasis, attention deficit-hyperactivity disorder	No significant impact	No significant impact	No significant impact
	N(alpha)-acetyltransferase 16, NatA Auxiliary Subunit (NAA16)	Enables ribosome binding activity. Involved in N-terminal protein amino acid acetylation; negative regulation of apoptotic process; and protein stabilization. Located in cytosol. Part of NatA complex. [provided by Alliance of Genome Resources, Apr 2022]	N/A	Hyperactivity	No significant impact	No significant impact
	Mitochondrial Translational Release Factor 1 (MTRF1)	The protein encoded by this gene was determined by in silico methods to be a mitochondrial protein with similarity to the peptide chain release factors (RFs) discovered in bacteria and yeast. The peptide chain release factors direct the termination of translation in response to the peptide chain termination codons. Initially thought to have a role in the termination of mitochondria protein synthesis, a recent publication found no mitochondrial translation release functionality. Multiple alternatively spliced transcript variants have been suggested by mRNA and EST data; however, their full-length natures are not clear. [provided by RefSeq, Jul 2008]	Alcoholic gastritis, spastic ataxia, aminoglycoside-induced deafness, combined oxidative phosphorylation deficiency 1, cox deficiency, infantile mitochondrial myopathy.	No significant impact	No significant impact	No significant impact
	Kelch Repeat and BTB Domain Containing 6 (KBTBD6)	Involved in proteasome-mediated ubiquitin-dependent protein catabolic process; protein K48-linked ubiquitination; and regulation of Rac protein signal transduction. Located in cytoplasm and nucleus. Part of Cul3-RING ubiquitin ligase complex. [provided by Alliance of Genome Resources, Apr 2022]	N/A	Not tested	Not tested	Not tested
	WW Domain Binding Protein 4 (WBP4)	This gene encodes WW domain-containing binding protein 4. The WW domain represents a small and compact globular structure that interacts with proline-rich ligands. This encoded protein is a general spliceosomal protein that may play a role in cross-intron bridging of U1 and U2 snRNPs in the spliceosomal complex A. [provided by RefSeq, Jul 2008]	N/A	Not tested	Not tested	Not tested

Trait	Candidate Gene Name	NCBI Gene Summary	Disorders	IMPC Phenotypes (Behaviour/ Neurological)	IMPC Phenotypes (Nervous System)	IMPC Phenotypes (Vison/ Eye)
	E74 Like ETS Transcription Factor 1 (ELF1)	This gene encodes an E26 transformation-specific related transcription factor. The encoded protein is primarily expressed in lymphoid cells and acts as both an enhancer and a repressor to regulate transcription of various genes. Alternative splicing results in multiple transcript variants. [provided by RefSeq, Feb 2009]	Cd3zea deficiency, retinoblastoma, systemic lupus erythematosus, t-cell acute lymphoblastic leukaemia, immune deficiency disease	Not tested	Not tested	Not tested
	SGT1 Homolog, MIS12 Kinetochore Complex Assembly Cochaperone (SUGT1)	This gene encodes a highly conserved nuclear protein involved in kinetochore function and required for the G1/S and G2/M transitions. This protein interacts with heat shock protein 90. Alternative splicing results in multiple transcript variants. Pseudogenes for this gene have been defined on several different chromosomes. [provided by RefSeq, Mar 2016]	N/A	No significant impact	No significant impact	No significant impact
	Chondromodulin (CNMD)	This gene encodes a glycosylated transmembrane protein that is cleaved to form a mature, secreted protein. The N-terminus of the precursor protein shares characteristics with other surfactant proteins and is sometimes called chondrosurfactant protein although no biological activity has yet been defined for it. The C-terminus of the precursor protein contains a 25 kDa mature protein called leukocyte cell-derived chemotaxin-1 or chondromodulin-1. The mature protein promotes chondrocyte growth and inhibits angiogenesis. This gene is expressed in the avascular zone of prehypertrophic cartilage and its expression decreases during chondrocyte hypertrophy and vascular invasion. The mature protein likely plays a role in endochondral bone development by permitting cartilaginous anlagen to be vascularized and replaced by bone. It may be involved also in the broad control of tissue vascularization during development. Alternative splicing results in multiple transcript variants encoding different isoforms. [provided by RefSeq, Jul 2008]	Infective endocarditis, chondrosarcoma, heart valve disease, aortic valve disease, pleomorphic adenoma	Not tested	Not tested	Not tested
	Protocadherin 8 (PCDH8)	This gene encodes a member of the CHMP/Chmp family of proteins which are involved in multivesicular body sorting of proteins to the interiors of lysosomes. The initial prediction of the protein sequence encoded by this gene suggested that the encoded protein was a metallopeptidase. The nomenclature has been updated recently to reflect the correct biological function of this encoded protein. Several transcripts encoding different isoforms have been found for this gene. [provided by RefSeq, Dec 2012]	Pontocerebellar hypoplasia, quantitative or qualitative defects of alpha-dystroglycanopathy, lissencephaly	No significant impact	No significant impact	No significant impact
	Olfactomedin 4 (OLFM4)	The protein encoded by this gene is a transcriptional activator, having been shown to increase the transcription of activator protein-1 and serum response element. The encoded protein can also form a complex with KBTBD6 and CUL3, which regulates the ubiquitylation and degradation of TIAM1, which is a regulator of RAC1. [provided by RefSeq, Jul 2016]	N/A	No significant impact	No significant impact	No significant impact
	Kelch Repeat and BTB Domain Containing 7 (KBTBD7)	The protein encoded by this gene is a transcriptional activator, having been shown to increase the transcription of activator protein-1 and serum response element. The encoded protein can also form a complex with KBTBD6 and CUL3, which regulates the ubiquitylation and degradation of TIAM1, which is a regulator of RAC1. [provided by RefSeq, Jul 2016]	N/A	Abnormal behaviour, decreased grip strength, decreased thigmotaxis, hyperactivity	No significant impact	No significant impact

Trait	Candidate Gene Name	NCBI Gene Summary	Disorders	IMPC Phenotypes (Behaviour/ Neurological)	IMPC Phenotypes (Nervous System)	IMPC Phenotypes (Vison/ Eye)
	Epithelial Stromal Interaction 1 (EPSTI1)	The protein encoded by this gene has been shown to promote tumor invasion and metastasis in some invasive cancer cells when overexpressed. Expression of this gene has been shown to be upregulated by direct binding of the Kruppel like factor 8 protein to promoter sequences. The translated protein interacts with the amino terminal region of the valosin containing protein gene product, resulting in the nuclear translocation of the nuclear factor kappa B subunit 1 gene product, and activation of target genes. Overexpression of this gene has been observed in some breast cancers and in some individuals with systemic lupus erythematosus (SLE). [provided by RefSeq, Sep 2016]	Lupus erythematous, breast cancer, Potocki-Shaffer Syndrome	Not tested	Not tested	Not tested
Trainability	**Reactive Oxygen Species Modulator 1 (ROMO1)**	The protein encoded by this gene is a mitochondrial membrane protein that is responsible for increasing the level of reactive oxygen species (ROS) in cells. The protein also has antimicrobial activity against a variety of bacteria by inducing bacterial membrane breakage. [provided by RefSeq, Nov 2014]	Premature ovarian failure, optic atrophy with or without deafness, ophthalmoplegia, myopathy, ataxia and neuropathy.	Hyperactivity	No significant impact	No significant impact
	**Adhesion G Protein-Coupled Receptor L2 (ADGRL2)**	This gene encodes a member of the latrophilin subfamily of G-protein coupled receptors. The encoded protein participates in the regulation of exocytosis. The proprotein is thought to be further cleaved within a cysteine-rich G-protein-coupled receptor proteolysis site into two chains that are non-covalently bound at the cell membrane. Alternative splicing results in multiple transcript variants. [provided by RefSeq, Jul 2014]	Encephalitozoonosis, eosinophilic meningitis, 3-methylglutaconic aciduria, cortical dysplasia complex with other brain malformations, Usher Syndrome.	Not tested	Not tested	Not tested
	**Inositol 1,4,5-triphosphate Receptor 2 (ITPR2)**	The protein encoded by this gene belongs to the inositol 1,4,5-triphosphate receptor family, whose members are second messenger intracellular calcium release channels. These proteins mediate a rise in cytoplasmic calcium in response to receptor activated production of inositol triphosphate. Inositol triphosphate receptor-mediated signaling is involved in many processes including cell migration, cell division, smooth muscle contraction, and neuronal signaling. This protein is a type 2 receptor that consists of a cytoplasmic amino-terminus that binds inositol triphosphate, six membrane-spanning helices that contribute to the ion pore, and a short cytoplasmic carboxy-terminus. A mutation in this gene has been associated with anhidrosis, suggesting that intracellular calcium release mediated by this protein is required for eccrine sweat production. [provided by RefSeq, Apr 2015]	Anhidrosis, icthydrosis, frontotemporal dementia and/or amyotrophic lateral sclerosis.	Not tested	Not tested	Not tested
	Serine/Threonine Kinase 38 Like (STK38L)	Enables ATP binding activity; magnesium ion binding activity; and protein serine/threonine kinase activity. Involved in intracellular signal transduction. Acts upstream of or within protein phosphorylation. Located in cytosol. [provided by Alliance of Genome Resources, Apr 2022]	Bardet-Biedl Syndrome	Not tested	Not tested	Not tested
	Transmembrane 7 Superfamily Member 3 (TM7SF3)	Involved in cellular response to unfolded protein; negative regulation of programmed cell death; and positive regulation of insulin secretion. Located in plasma membrane. [provided by Alliance of Genome Resources, Apr 2022]	Childhood central nervous system embryonal tumour, cerebral dysgenesis, neuropathy, ichthyosis, palmoplantar keratoderma syndrome, microphthalmia, Kallman Syndrome, Warburg Micro Syndrome	Not tested	Not tested	Not tested

Trait	Candidate Gene Name	NCBI Gene Summary	Disorders	IMPC Phenotypes (Behaviour/ Neurological)	IMPC Phenotypes (Nervous System)	IMPC Phenotypes (Vison/ Eye)
	FGFR1 Oncogene Partner 2 (FGFR1OP2)	Predicted to enable identical protein binding activity. Predicted to be involved in response to wounding. Predicted to act upstream of or within wound healing. Predicted to be located in cytoplasm. [provided by Alliance of Genome Resources, Apr 2022]	Lymphoblastic lymphoma, myeloid and lymphoid neoplasms associated with Fgfr1 abnormalities, myeloproliferative neoplasm, leukaemia, acute myeloid	No significant impact	No significant impact	Significant impact
	Integrator Complex Subunit 13 (INTS13)	Involved in regulation of mitotic cell cycle. Acts upstream of or within centrosome localization; mitotic spindle organization; and protein localization to nuclear envelope. Located in cytoplasm and nuclear body. [provided by Alliance of Genome Resources, Apr 2022]	Orofaciodigital Syndrome	Not tested	Not tested	Not tested
	Mediator Complex Subunit 21 (MED21)	This gene encodes a member of the mediator complex subunit 21 family. The encoded protein interacts with the human RNA polymerase II holoenzyme and is involved in transcriptional regulation of RNA polymerase II transcribed genes. A pseudogene of this gene is located on chromosome 8. Alternative splicing results in multiple transcript variants. [provided by RefSeq, Nov 2012]	Teratoma with somatic-type malignancies, hemopericardium , hemopneumothorax, Opitz-Kaveggia Syndrome.	Not tested	Not tested	Not tested

Table S5. Summary of minimal model and regression coefficients for each of the CBARQ behavior traits. The first column (CBARQ Traits) identifies the behavioral trait analyzed. The second column (Minimal Model) lists the predictor retained in the minimal model for that trait. The subsequent columns provide the coefficient estimates (Coefficient), the 95% confidence intervals (Confidence Interval), and the associated p-values (P value) for each predictor. The final column (GWAS Covariates) specifies the covariates used in each trait's genome-wide association study (GWAS) analyses based on significant predictors. For quantitative traits, coefficients represent the change in the outcome per unit change of the predictor; for binary traits, coefficients are expressed in log-odds. P-values below 0.05 are marked with an asterisk (*).

CBARQ Traits	Minimal Model	Coefficient	Confidence Interval	P_value	GWAS Covariates
**Attachment Attention Seeking**	sex	0.098	[0.018, 0.179]	0.0167*	sex
	any_disease	0.068	[-0.024, 0.16]	0.1452	
	activity_level	0.062	[-0.015, 0.139]	0.1168	
**Chasing**	age	-0.058	[-0.107, -0.008]	0.0238*	age + activity_level
	sex	-0.107	[-0.216, 0.001]	0.0532	
	activity_level	0.211	[0.107, 0.315]	<0.001*	
**Dog Directed Aggression**	age	0.152	[-0.024, 0.328]	0.0908	sex + activity_level
	sex	0.908	[0.519, 1.306]	<0.001*	
	activity_level	0.647	[0.271, 1.028]	<0.001*	
**Dog Directed Fear**	neuter_status	1.128	[0.5, 1.846]	<0.001*	neuter_status
	service_dog	-14.722	[NA, 68.705]	0.984	
**Dog Rivalry**	activity_level	0.218	[-0.076, 0.512]	0.145	None
	service_dog	-14.167	[NA, 15.812]	0.973	
**Energy**	activity_level	0.979	[0.706, 1.254]	<0.001*	activity_level
**Excitability**	sex	0.147	[0.06, 0.234]	<0.001*	sex + neuter_status + activity_level
	neuter_status	0.148	[0.031, 0.265]	0.0132*	
	activity_level	0.106	[0.023, 0.188]	0.0119*	
**Nonsocial Fear**	age	-0.205	[-0.398, -0.015]	0.03630*	age + neuter_status + any_disease + activity_level
	neuter_status	1.062	[0.46, 1.725]	< 0.001*	
	any_disease	0.737	[0.244, 1.255]	0.00418*	
	activity_level	-0.559	[-0.994, -0.138]	0.01030*	
**Owner Directed Aggression**	any_disease	0.387	[-0.07, 0.872]	0.106	sex
	sex	0.465	[0.068, 0.87]	0.0229*	
	service_dog	1.282	[-0.423, 2.986]	0.1212	
	activity_level	0.344	[-0.015, 0.704]	0.0604	
**Stranger Directed Fear**	age	-0.209	[-0.371, -0.049]	0.01091*	age + sex
	sex	-0.472	[-0.83, -0.12]	0.00901*	
	neuter_status	0.431	[-0.078, 0.985]	0.11039	
**Touch Sensitivity**	neuter_status	0.842	[0.292, 1.455]	0.00432*	neuter_status + activity_level
	any_disease	0.304	[-0.111, 0.738]	0.15887	
	activity_level	-0.38	[-0.738, -0.03]	0.03510*	
**Trainability**	age	-0.021	[-0.037, -0.005]	0.0103*	age + activity_level
	activity_level	0.048	[0.014, 0.082]	0.0053*	
	service_dog	0.148	[-0.021, 0.317]	0.0869	

Table S6. Canine genes identified in the C-BARQ GWAS had multiple significant PheWAS associations for human temperamental and cognitive traits. The proximal candidate genes identified at loci significantly associated with C-BARQ factor scores in the canine GWAS were interrogated for association with human temperamental and cognitive traits; PMID, PubMed identifier of relevant human GWAS study; Year, of publication; p-value, n, significance and number of subjects in the human GWAS.

C-BARQ Factor	Canine gene	Human traits	P-value	N	Year	PMID
**Dog-directed fear**	*ZC3H12C*	Educational attainment	2.23E-05	766345	2018	30038396
	PRDX1	Educational attainment	5.32E-05	766345	2018	30038396
		Mania - Ever had period extreme irritability	1.12E-04	122891	2019	31427789
	HUNK	Educational attainment	8.27E-07	766345	2018	30038396
		Worry too long after embarrassment	1.37E-04	370660	2019	31427789
		Ever smoked	6.05E-05	385013	2019	31427789
**Non-social fear**	*ASCC3*	Mood swings	1.51E-04	377179	2019	31427789
		Miserableness	5.26E-05	379907	2019	31427789
		Irritability	1.66E-07	369232	2019	31427789
		Sensitivity / hurt feelings	5.03E-05	375272	2019	31427789
		Seen doctor (GP) for nerves, anxiety, tension or depression	3.35E-05	383771	2019	31427789
		Neuroticism score	3.88E-05	312740	2019	31427789
		Anxiety - Recent worrying too much about different things	2.46E-04	126284	2019	31427789
		Depression - Waking too early	1.42E-04	48926	2019	31427789
		Neuroticism	1.88E-08	390278	2018	29942085
		Depressive affect subcluster	1.63E-06	357957	2018	29942085
		Neuroticism sum score	3.46E-08	380506	2018	29500382
		Mood swings (MOOD)	1.32E-04	265382	2018	29500382
		Miserableness (MIS)	2.00E-05	267050	2018	29500382
		Irritability (IRR)	4.19E-08	260369	2018	29500382
		Sensitivity / hurt feelings (HURT)	1.94E-04	264144	2018	29500382
		Neuroticism	5.48E-08	329821	2018	29255261
		Loneliness (MTAG)	1.21E-05	487647	2018	29970889

		Depression	1.34E-05	500199	2019	30718901
		Neuroticism (univariate)	4.84E-06	523783	2019	30643256
		Depressive symptoms (univariate)	3.14E-06	1067913	2019	30643256
		Neuroticism (MA GWAMA)	2.24E-07	523783	2019	30643256
		Depressive symptoms (MA GWAMA)	9.12E-07	1067913	2019	30643256
		Well-being spectrum	4.97E-09	2311184	2019	30643256
**Stranger-directed fear**	*ADD2*	Depression - Professional informed about depression	4.06E-05	71538	2019	31427789
**Dog-directed aggression**	*PTPN1*	Intelligence	6.09E-05	269867	2018	29942086
		Cognitive performance	3.82E-06	257828	2018	30038396
		Educational attainment	8.30E-07	766345	2018	30038396
		Major depressive disorder	2.54E-08	173005	2018	29700475
**Trainability**	*ROMO1*	Fluid intelligence score	1.55E-06	125935	2019	31427789
		Intelligence	1.66E-08	269867	2018	29942086
		Cognitive performance	2.36E-06	257828	2018	30038396
		Depression - Recent feelings of depression	5.77E-05	126384	2019	31427789
	ADGRL2 (LPHN2)	Intelligence	6.43E-06	269867	2018	29942086
		Irritability	4.11E-05	369232	2019	31427789
		Sensitivity / hurt feelings	1.08E-04	375272	2019	31427789
		Guilty feelings	1.90E-05	376361	2019	31427789
		Irritability (IRR)	1.13E-04	260369	2018	29500382
		Sensitivity / hurt feelings (HURT)	2.45E-05	264144	2018	29500382
		Guilty feelings (GUILT)	9.72E-06	265139	2018	29500382
		Educational attainment	1.51E-06	766345	2018	30038396
	ITPR2	Worry too long after embarrassment	1.39E-04	370660	2019	31427789
		Seen a psychiatrist for nerves, anxiety, tension or depression	7.97E-05	384700	2019	31427789

		Worry too long after embarrassment (WORR-EMB)	1.76E-04	261094	2018	29500382
		Well-being spectrum	4.69E-05	2311184	2019	30643256
**Touch sensitivity**	*SLC35F6*	Risk taking	1.30E-05	372651	2019	31427789
		Sleep duration	5.75E-05	384317	2019	30804565
		Schizophrenia	1.50E-05	105318	2018	29483656
		Schizophrenia/Bipolar disorder	1.13E-04	107620	2018	29906448
	VWA8	Educational attainment	2.24E-04	766345	2018	30038396
**Separation related problem**	*IGSF11*	Educational attainment	5.05E-05	766345	2018	30038396

Dataset S1 (separate file)

Significant GWAS for C-BARQ Traits

Results in sheet 1 (quantitative traits) shows variants that surpassed the significance threshold ($p < 1\times10^{-5}$) in the analysis of continuous behavioral traits. For each association, the table reports the chromosome (CHR), SNP identifier (SNP), base pair position (BP), allele frequency (AF), effect allele (ALLEL1), reference allele (ALLEL0), and the beta coefficient (BETA) with its standard error (SE) and corresponding lower (BETA_95CI_LOWER) and upper (BETA_95CI_UPPER) 95% confidence interval. The (P value) represent the p-value, which indicates the level of statistical significance for the association, and the Trait column specifies the behavioral phenotype analyzed as quantitative traits. Result in sheet 2 (binary trait) shows variants that surpassed the significance threshold ($p < 1\times10^{-5}$) in the analysis of dichotomous behavioral traits. For each association, the table reports the chromosome (CHR), SNP identifier (SNP), base pair position (BP), allele frequency (AF), effect allele (ALLEL1), reference allele (ALLEL0), and the odds ratio (OR) with its corresponding lower (BETA_95CI_LOWER) and upper (BETA_95CI_UPPER) 95% confidence interval. The (P value) represent the p-value, which indicates the level of statistical significance for the association, and the Trait column specifies the behavioral phenotype analyzed as binary trait.

Dataset S2 (separate file)

PheWAS Associations of Human Orthologs for Candidate Genes from Canine GWAS for C-BARQ. This table lists the human temperament and cognitive related traits derived from GWAS studies with N ≥ 45,000. The ATLAS ID is the trait identifier from Atlas of Complex Trait Genetics (ATG) database and corresponding human temperament and cognitive related traits description and study year in bracket.

Dataset S3 (separate file)

Canine genes identified in the C-BARQ GWAS had no significant PheWAS associations for unrelated human psychiatric, temperamental and cognitive traits. The proximal candidate genes identified at loci significantly associated with C-BARQ factor scores in the canine GWAS were interrogated for association with human psychiatric, temperamental and cognitive traits; PMID, PubMed identifier of relevant human GWAS study; Year, of publication; Domain, General domain of the trait; Chapter level, chapter of the trait obtained from either ICD10 or ICF10; Subchapter level, Subchapter of the trait obtained from either ICD10 or ICF10; Trait, The trait name used in the original study (or as close as possible); uniqTrait, the trait name harmonized across database; Population, one of the five super ancestry populations defined in 1000 genome project; N, total number of sample size used for the analyses.

Supplementary Information References

1. S. Padmanabhan, *et al.*, Genome-wide association study of blood pressure extremes identifies variant near UMOD associated with hypertension. *PLoS Genet* **6**, e1001177 (2010).

2. Y. Li, *et al.*, Extreme sampling design in genetic association mapping of quantitative trait loci using balanced and unbalanced case-control samples. *Sci Rep* **9**, 15504 (2019).

3. T. Bjørnland, A. Bye, E. Ryeng, U. Wisløff, M. Langaas, Powerful extreme phenotype sampling designs and score tests for genetic association studies. *Statistics in Medicine* **37**, 4234–4251 (2018).

4. W. Zhou, *et al.*, Efficiently controlling for case-control imbalance and sample relatedness in large-scale genetic association studies. *Nat Genet* **50**, 1335–1341 (2018).

5. H. Chen, *et al.*, Control for Population Structure and Relatedness for Binary Traits in Genetic Association Studies via Logistic Mixed Models. *The American Journal of Human Genetics* **98**, 653–666 (2016).

6. X. Dai, G. Fu, S. Zhao, Y. Zeng, Statistical Learning Methods Applicable to Genome-Wide Association Studies on Unbalanced Case-Control Disease Data. *Genes* **12**, 736 (2021).

7. P.-R. Loh, *et al.*, Efficient Bayesian mixed-model analysis increases association power in large cohorts. *Nat Genet* **47**, 284–290 (2015).

8. J. Yang, S. H. Lee, M. E. Goddard, P. M. Visscher, GCTA: A Tool for Genome-wide Complex Trait Analysis. *AJHG* **88**, 76 (2011).

9. P. J. Van der Most, L. K. Küpers, H. Snieder, I. Nolte, QCEWAS: automated quality control of results of epigenome-wide association studies. *Bioinformatics* **33**, 1243–1245 (2017).

10. L. Lloyd-Jones, M. Robinson, J. Yang, P. Visscher, Transformation of Summary Statistics from Linear Mixed Model Association on All-or-None Traits to Odds Ratio. *Genetics* **208**, 1397–1408 (2018).

11. Ovid MEDLINE®. Available at: https://www-wolterskluwer-com.ezp.lib.cam.ac.uk/en/solutions/ovid/ovid-medline-901 [Accessed 7 October 2024].

12. M. Mahmoodi, A. Mehrgardi, M. Momen, J. Serpell, Deciphering the genetic basis of behavioral traits in dogs: Observed-trait GWAS and latent-trait GWAS analysis reveal key genes and variants. *Vet J* **308**, 106251 (2024).

13. S. Mastrangelo, *et al.*, Genome-wide association study for morphological and hunting-behavior traits in Braque Franc¸ais Type Pyrenees dogs: A preliminary study. *The Veterinary Journal* **306**, 106189 (2024).

14. A. W. Eyre, *et al.*, Genome scanning of behavioral selection in a canine olfactory detection breeding cohort. *Sci Rep* **12**, 1–12 (2022).

15. S. Shan, F. Xu, B. Brenig, Genome-Wide Association Studies Reveal Neurological Genes for Dog Herding, Predation, Temperament, and Trainability Traits. *Front. Vet. Sci.* **8** (2021).

16. Y. Matsumoto, A. Konno, G. Ishihara, M. Inoue-Murayama, Genetic dissection of behavioral traits related to successful training of drug detection dogs. *Sci Rep* **13**, 1–10 (2023).

17. J. Friedrich, *et al.*, Genetic dissection of complex behaviour traits in German Shepherd dogs. *Heredity (Edinb)* **123**, 746–758 (2019).

18. J. Friedrich, *et al.*, Unravelling selection signatures in a single dog breed suggests recent selection for morphological and behavioral traits. *Adv Genet (Hoboken)* **1**, e10024 (2020).

19. J. Ilska, *et al.*, Genetic Characterization of Dog Personality Traits. *Genetics* **206**, 1101–1111 (2017).

20. I. Zapata, J. Serpell, C. Alvarez, Genetic mapping of canine fear and aggression-Web of Science Core Collection. *BMC Genomics* **17** (2016).

21. E. L. MacLean, N. Snyder-Mackler, B. M. vonHoldt, J. A. Serpell, Highly heritable and functionally relevant breed differences in dog behaviour. *Proc Biol Sci* **286**, 20190716 (2019).

22. Y. Hsu, J. Serpell, Development and validation of a questionnaire for measuring behavior and temperament traits in pet dogs. *JAVMA-J. Am. Vet. Med. Assoc.* **223**, 1293–300 (2003).

23. G. Stelzer, *et al.*, The GeneCards Suite: From Gene Data Mining to Disease Genome Sequence Analyses. *Curr Protoc Bioinformatics* **54**, 1.30.1-1.30.33 (2016).

24. T. Groza, *et al.*, The International Mouse Phenotyping Consortium: comprehensive knockout phenotyping underpinning the study of human disease. *Nucleic Acids Res* **51**, D1038–D1045 (2023).
